# Structural basis for Rad54- and Hed1-mediated regulation of Rad51 during the transition from mitotic to meiotic recombination

**DOI:** 10.1073/pnas.2510007122

**Published:** 2025-09-11

**Authors:** Yeonoh Shin, Michael T. Petassi, Aidan M. Jessop, Stefan Y. Kim, Razvan Matei, Katherine Morse, Vivek B. Raina, Upasana Roy, Eric C. Greene

**Affiliations:** ^a^Department of Biochemistry & Molecular Biophysics, Columbia University Irving Medical Center, New York, NY 10032

**Keywords:** homologous recombination, Rad51, meiosis, Rad54, Hed1

## Abstract

Rad51 catalyzes the DNA pairing reactions that take place during homologous recombination (HR), and HR must be tightly regulated to ensure physiologically appropriate outcomes. Rad54 is an ATP-dependent DNA motor protein that stimulates Rad51 activity during mitosis. In meiosis Rad51 is downregulated by the protein Hed1, which blocks Rad54 binding to Rad51, and allows Dmc1 to function as the active recombinase. We currently have a poor understanding of the regulatory interplay between Rad54, Hed1, Rad51, and Dmc1. Here, we identify a conserved Rad51 interaction motif within Rad54, and we solve a CryoEM structure of this motif bound to Rad51. We also identify a distinct Rad51 interaction motif within Hed1 and solve its structure bound to Rad51. These structures explain how Rad54 engages Rad51 to promote recombination between sister chromatids during mitosis and how Rad51 is downregulated by Hed1 upon entry into meiosis such that its meiosis-specific homolog Dmc1 can promote recombination between homologous chromosomes.

## Significance

Rad51 is a protein that helps repair broken DNA through a process called homologous recombination. This process must be carefully regulated so it happens at the right time and place. During mitosis, another protein called Rad54 helps Rad51 do its job. But during meiosis, Rad51 gets turned off by a protein called Hed1, so that a related protein named Dmc1 can take over. We do not fully understand how these proteins work together, but in this study, we have determined how Rad54 and Hed1 each bind to Rad51. Using cryogenic electron microscopy, we have solved the structures that explain how Rad54 helps Rad51 during regular cell division, and how Hed1 blocks Rad51 during meiosis so Dmc1 can take over.

Homologous recombination (HR) is a conserved pathway used to repair double strand breaks (DSB), repair stalled and collapsed replication forks, and promote proper chromosome segregation and enhance genetic diversity during meiosis (1–3). HR is essential for maintaining genome integrity and defects in HR are broadly associated with cancers and cancer-prone syndromes (4, 5).

The Rad51/RecA family of proteins plays a central role in catalyzing the DNA pairing reactions that take place during HR (4, 6, 7). Rad51 is an ATP-dependent DNA-binding protein that forms extended right-handed helical filaments on the 3′ single-stranded DNA (ssDNA) overhangs that are formed following end processing of DSBs (1, 2, 7–11). The resulting Rad51-ssDNA nucleoprotein filament is also referred to as the presynaptic complex (1, 2, 12). The Rad51 presynaptic complex is responsible for identifying a homologous double-stranded DNA (dsDNA) sequence that can be used as a template to guide repair of the damaged DNA, and it catalyzes a strand invasion reaction where it pairs the bound ssDNA with the complementary strand of the homologous dsDNA, resulting in displacement of the non–complementary DNA strand. The resulting D-loop intermediate can be processed through several different pathways, leading to repair of the damaged DNA (1, 2, 12–14).

Rad54 is a highly conserved member of the Swi/Snf family of ATP-dependent dsDNA translocases (7, 15–17). *RAD54* deletion imparts sensitivity to DNA damaging agents (18, 19), causes defects in strand invasion (20, 21), leads to the accumulation of toxic HR intermediates (22), and *rad54* missense mutations have been linked to human cancers (23, 24). Rad54 binds to the Rad51 presynaptic complex, drives the ATP-hydrolysis-dependent movement of the presynaptic complex along dsDNA during the search for sequence homology, and it greatly increases the efficiency of Rad51-mediated strand invasion (7, 25–30). Rad54 has also been implicated in the stabilization of the Rad51 presynaptic complex (31, 32), DNA branch migration (33–35), nucleosome remodeling (36–38), and the removal of Rad51 from dsDNA (17, 39, 40).

The transition from mitotic to meiotic recombination involves a change in repair template preference: in mitosis, recombination occurs preferentially between sister chromatids, while in meiosis, it takes place more frequently between homologous chromosomes (41–43). Meiotic recombination also involves the selective downregulation of Rad51 activity, allowing the meiosis-specific recombinase Dmc1 to catalyze strand exchange (3, 41, 44–48). Therefore, Dmc1 is the active recombinase during meiosis, with Rad51 acting as an accessory factor to facilitate Dmc1 filament assembly (41, 44). Dmc1-mediated repair of the programmed DSBs that are generated by Spo11 during meiosis creates physical linkages between homologous chromosomes called chiasma, which are essential to ensure proper chromosome segregation (41, 43, 49, 50). In *Saccharomyces cerevisiae*, Rad51 inhibition is achieved through two meiosis-specific regulatory proteins, Mek1 and Hed1 (41, 51). Mek1 is a kinase that phosphorylates Rad54 to weaken its interaction with Rad51 (51) and Hed1 is a regulatory protein that binds to Rad51 and blocks the association of Rad54 (52, 53). Rad54 is a required cofactor for Rad51 strand invasion activity, therefore, Hed1 binding downregulates the activity of Rad51 in meiosis by preventing interactions between Rad51 and Rad54. Interestingly, even though *S. cerevisiae* Dmc1 and Rad51 both interact with Rad54, the negative regulatory function of Hed1 is highly specific for just Rad51 (53–55).

We have a limited understanding of the mechanistic interplay that underlies the regulation of Rad51 by cofactors such as Rad54 and Hed1 largely because there are no high-resolution structures of Rad51 presynaptic complexes bound to these cofactors. Here, we use a combination of bioinformatics and AlphaFold3 modeling to identify a broadly conserved peptide within the unstructured N-terminal domain (NTD) of Rad54 that is predicted to interact with Rad51. We then solve the CryoEM structure of this Rad51 interaction motif bound to the Rad51-ssDNA filament and use a combination of deep mutagenesis and site-directed mutagenesis to validate important amino acid residue contacts, providing structural insights into Rad54 protein–protein interactions with the Rad51 presynaptic complex. We use a similar approach to predict how Hed1 binds to Rad51, we then solve the CryoEM structure of the Rad51 interaction motif from Hed1 bound to a Rad51-ssDNA filament and we validate important amino acid residues using mutagenesis. Our data demonstrate that the Hed1 binding pocket physically overlaps with the Rad54 binding site, explaining how Hed1 acts as a potent negative regulatory factor of Rad51 strand exchange activity during meiosis. Notably, although their binding sites overlap, the binding mechanisms of Rad54 and Hed1 are completely different from one another. These distinct binding mechanisms help to explain how Rad54 can interact with both Rad51 and Dmc1, whereas Hed1 selectively binds to just Rad51 and does not bind to Dmc1, even though Rad51 and Dmc1 share 45% sequence identity, thus allowing Dmc1 to act as the primary recombinase for promoting recombination between homologous chromosomes during meiosis.

## Results

### Predicted Interaction between Rad54 and Rad51.

**Fig. 1.**
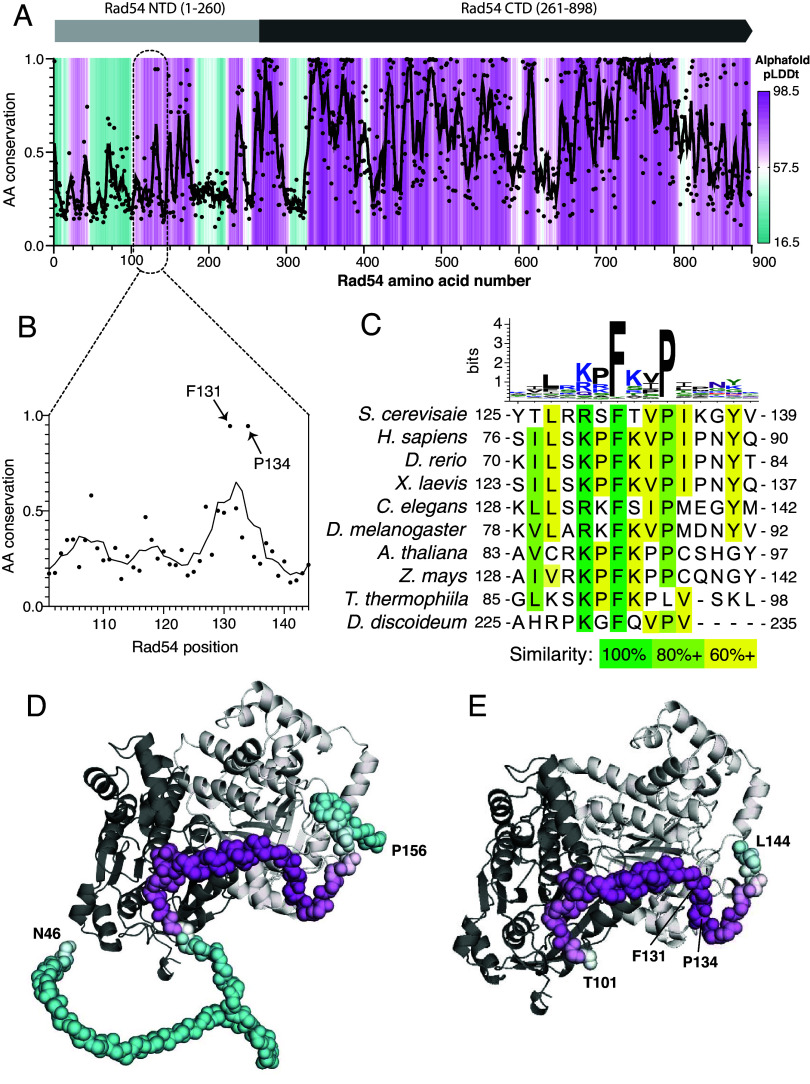
Predicted interaction interface between Rad51 and Rad54. (*A*) Plot of amino acid residue conservation and predicted AlphaFold3 structure (pLDDt; predicted local difference distance test) versus residue number for *S. cerevisiae* Rad54. Individual amino acid positions are indicated as black dots with a 5-amino acid moving average plotted as a black line. pLDDt is colored from cyan to purple as indicated by the scale bar. (*B*) Residue conservation in the region of the NTD FxxP motif. (*C*) Sequence logo and sequence alignments for the region encompassing the FxxP motif. (*D*) Predicted AlphaFold3 structure for a Rad54 residues N46 to P156 or (*E*) Rad54 residues T101 to L144 bound to two adjacent Rad51 monomers from within a Rad51 filament.

Crystal structures of the core Snf2 motor domain from *Danio rerio* Rad54 and a Rad54 homolog from *Sulfolobus solfataricus* have been reported (56, 57). However, there are no structures showing how Rad54 interacts with the presynaptic complex. Initial efforts at obtaining CryoEM structures of *S. cerevisiae* Rad51 bound to full-length *S. cerevisiae* Rad54 (898 aa) were not successful, therefore we sought to obtain a structure of a smaller complex that would still enable us to define how Rad54 interacts with the Rad51-ssDNA presynaptic filament. For this, we used a combination of bioinformatics and AlphaFold3 modeling to predict which region of Rad54 might be involved in direct contacts with Rad51 (Fig. 1). Rad54 is composed of a largely unstructured NTD that interacts with Rad51 and a C-terminal domain (CTD) containing an Snf2 ATPase motor domain which allows for dsDNA translocation activity that essential to protein function (Fig. 1*A*) (26, 58–62). Bioinformatic analysis revealed a conserved region (amino acids 101 to 144, Fig. 1*B*) within the Rad54 NTD that also contains an FxxP motif conserved across eukaryotes (Fig. 1*C*). As anticipated, AlphaFold3 predictions suggest that the Rad54 NTD is largely unstructured (*SI Appendix*, Fig. S1 *A* and *B*), consistent with previous studies (25). However, AlphaFold3 predictions also suggested that a portion of the NTD containing the FxxP motif becomes more highly structured in the presence of Rad51 and can interact with the Rad51-ssDNA nucleoprotein filament (Fig. 1 *D* and *E* and *SI Appendix*, Fig. S1 *C* and *D*). Given these considerations, we tentatively defined *S. cerevisiae* Rad54 amino acid residues T101 to L144 as a potential Rad51 interaction motif.

### Structure of the Rad54-Bound Presynaptic Complex.

**Fig. 2.**
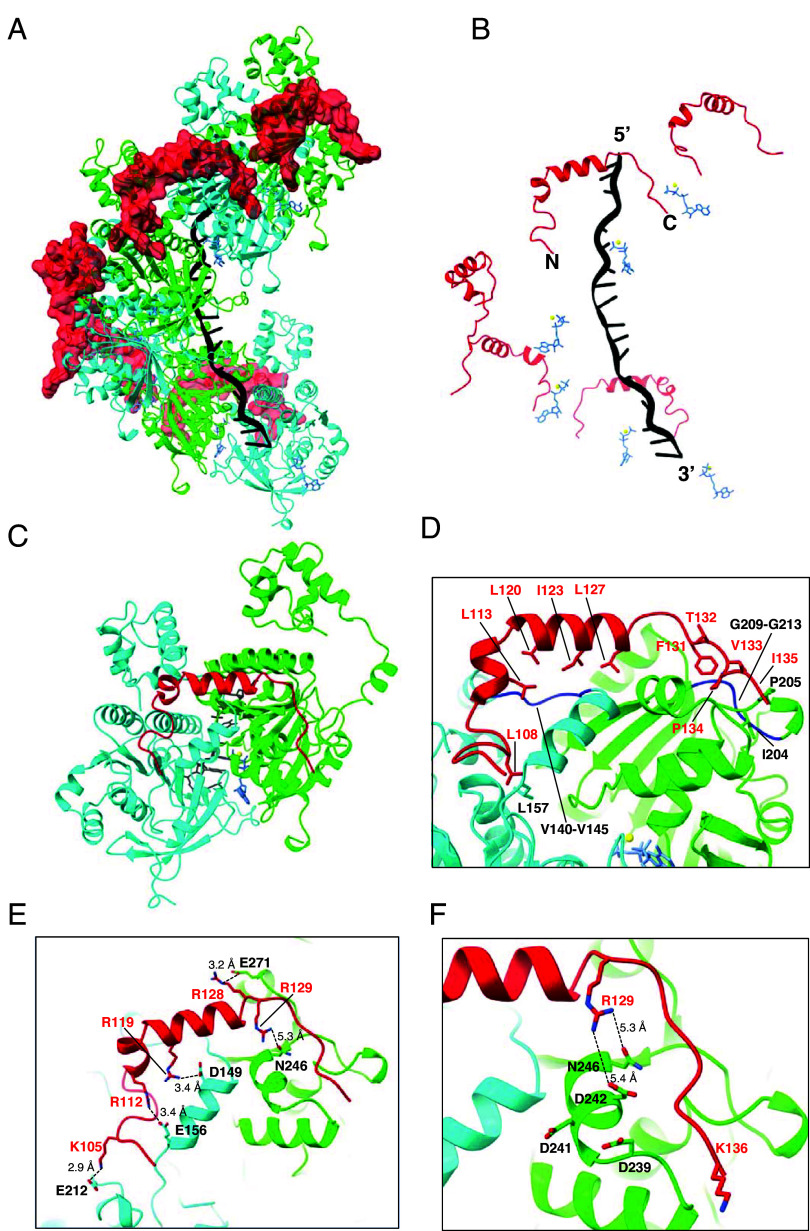
Structure of the Rad51 interaction motif of Rad54 bound to the Rad51-ssDNA filament. (*A*) CryoEM reconstruction of the Rad51-ssDNA filament bound by a peptide encompassing Rad54 residues T101 to L144. Rad51 subunits are shown in alternating colors (cyan and green) and the Rad54 peptide is shown in red. (*B*) Structure in which the Rad51 proteins have been removed to highlight the relative locations of the Rad54 peptide, Rad51-bound ssDNA, and ATP molecules. (*C*) Structure showing that the Rad54 peptide is bound to two adjacent Rad51 subunits (the 5′ oriented subunit is shown in green and the 3′ oriented subunit is shown in cyan). (*D*–*F*) Details of the molecular interactions between Rad54 (shown in red) and the two adjacent Rad51 subunits.

We obtained a 3.3 Å resolution CyroEM structure of an *S. cerevisiae* Rad51 filament bound to a 96-nucleotide ssDNA fragment in the presence of ATP along with a 44 amino acid peptide encompassing residues T101 to L144 from Rad54 (Fig. 2 and *SI Appendix*, Figs. S2 and S3 *A* and *B* and Table S1). The Rad54 peptide decorates the outside surface of the Rad51 filament, and it is located on the opposite side of the nucleoprotein filament relative to the bound strand of ssDNA (Fig. 2 *A* and *B*). Each Rad54 peptide interacts with a composite binding site formed by two adjacent Rad51 protomers within the nucleoprotein filament, corresponding to a buried surface area of 1651.4 Å^2^ (Fig. 2 *A* and *C*). The Rad54 peptide is composed of a N-terminal loop (amino acid residues R103 to A110), followed by a short helix (~1 turn; residues Q111 to D116), then another short helix (~3 turns; residues P117 to T126), and ends with a loop region (residues L127 to K136) that contains the FxxP motif and is located near the C-terminus of the peptide. Rad54 residues R103 to T126 interact with the Rad51 protomer facing toward the 3′ end of the ssDNA (shown in cyan; Fig. 2 *D*–*F*) and residues R128 to K136 interact with the 5′ facing Rad51 protomer (shown in green; Fig. 2 *D*–*F*). Close inspection of the structure reveals that the Rad54 peptide interacts with Rad51 primarily through a combination of hydrophobic and electrostatic contacts. There are two hydrophobic regions on the surface of the Rad51 filament that interact with the Rad54 peptide. Rad51 residues V140 to V145 form a hydrophobic pocket at the Rad51 protomer–protomer interface which interacts with leucine residues L113, L120, and L127 and isoleucine residues I123 from the Rad54 peptide (Fig. 2*D*). Rad51 residues G209 to G213 form a second hydrophobic pocket which interacts with the FxxP motif of Rad54 (Fig. 2*D*). Notably, these hydrophobic pockets are highly conserved in both the Rad51 and Dmc1 lineages of the Rad51/RecA family of proteins, suggesting that this may be a general mechanism for Rad54 binding to the surface of these two eukaryotic recombinases (*Discussion*). In addition to these hydrophobic contacts, the basic residues K105, R112, R119, R128, and R129 from Rad54 form a network of electrostatic interactions with Rad51 residues E212, E156, D149, E271, and N246 (Fig. 2*E*). For brevity, we will hereafter refer to the 44 amino acid residue peptide (T101 to L144) as the Rad51 interaction motif of Rad54.

### Analysis of the Rad51 Conserved Protruding Acidic Patch.

It has been proposed that a conserved protruding acidic patch (PAP) on the surface of Rad51, composed of residues D239, D241, and D242, was involved in interactions with Rad54 (63). Surprisingly, these PAP residues do not appear to form obvious strong interactions with the Rad51 interaction motif from the Rad54 peptide in our structure (Fig. 2*F*). Rad51 residue D241 faces away from the bound Rad54 peptide, and Rad51 residues D239 and D242 are too far from the nearest basic residues in Rad54 to form strong electrostatic interactions (Fig. 2*F*). To test the potential contributions of these acidic Rad51 residues to interactions with Rad54 we tested *rad51* mutants in which the PAP aspartic acid residues were changed to alanine and asked whether cells expressing these mutants could survive when grown on media containing the DNA damaging agent methyl methane sulfonate (MMS). The *rad51* single mutants (D239A; D241A; and D242A) and *rad51* double mutants (D241A, D242A; D239A, D241A; and D239A, D242A) all exhibit wild-type or near wild-type phenotypes when grown on media containing the DNA damaging agent MMS, whereas a *rad51* triple mutant (D239A, D241A, D242A) exhibits a null phenotype (*SI Appendix*, Fig. S3*C*). However, the triple mutant Rad51 protein still supports Rad54-dependent D-loop formation in vitro suggesting that the in vivo defect likely is not directly related to the disruption of Rad51 interactions with Rad54 (*SI Appendix*, Fig. S3*D*). We noted that the Rad51 triple mutant (D239A, D241A, D242A) precipitates during purification at salt concentrations lower than 500 mM NaCl, suggesting the possibility that in our genetic assays the three mutations may act to destabilize the protein rather than disrupting any specific interactions with Rad54. Alternatively, the triple mutant protein may be defective in cells due to disruption of interactions with other proteins such as Rad55-Rad57, Rad52, or both (63). Taken together, our data suggest that the Rad51 protruding acid patch might not be directly involved in contacts between Rad51 and Rad54.

### Genetic Analysis of the Rad51 Interaction Motif from Rad54.

**Fig. 3.**
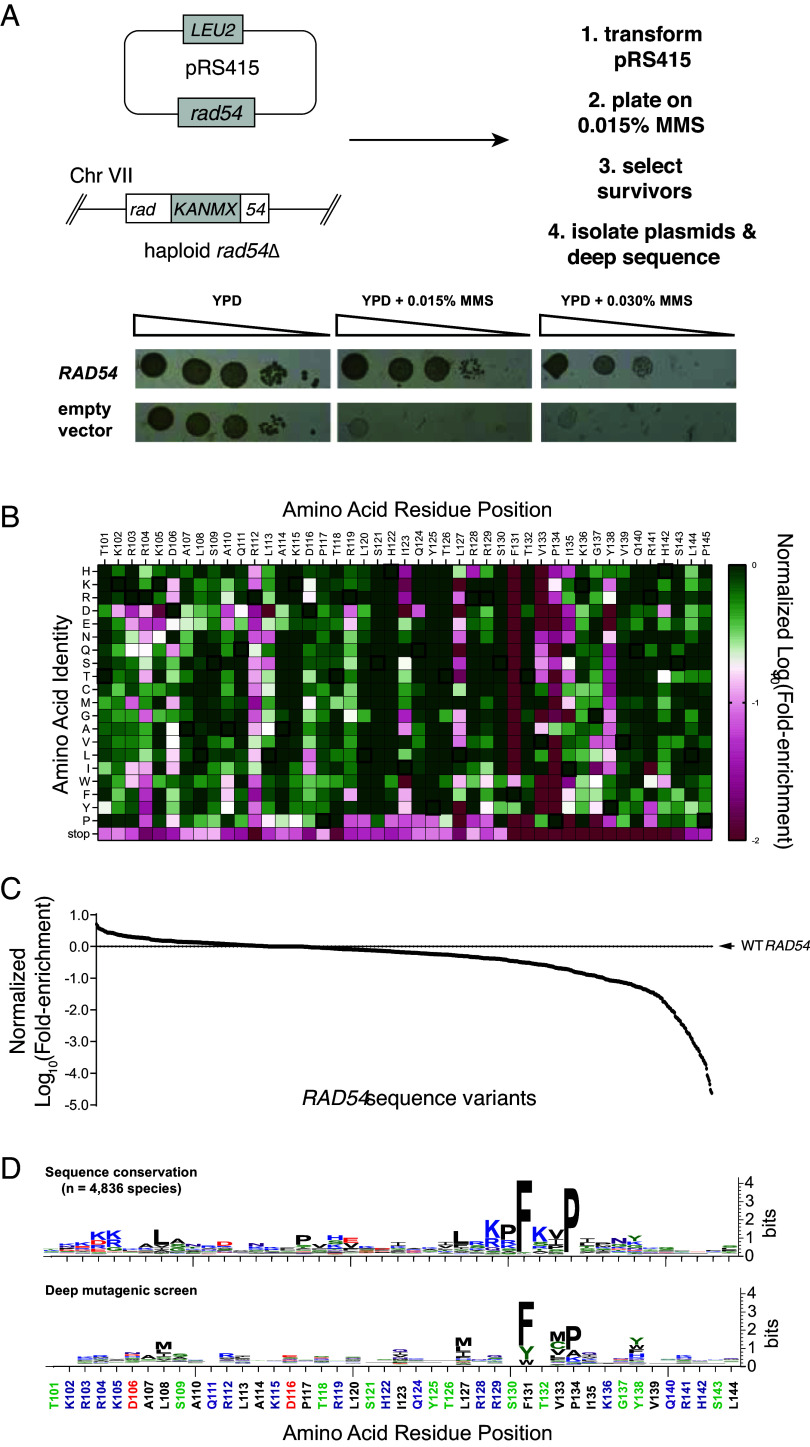
Functional landscape of the Rad54-Rad51 interface. (*A*) Strategy for deep mutagenic analysis of the Rad51 interaction motif from Rad54 and control spot assays on MMS-containing media for a *rad54Δ* strain complemented with either wild-type RAD54 or an empty vector. (*B*) Heat map showing Rad54 single mutant sequence variants that were recovered from the deep mutagenic screen. Data are normalized to the wild-type allele at each position and colored from green (corresponding to wild-type), to white (log_10_(−0.7), corresponding to ~fivefold depleted relative to wild-type), to red (log_10_(−2), corresponding to 100-fold depleted relative to wild-type). (*C*) Distribution of all data values for the deep mutagenic screen. (*D*) Sequence logos comparing the Rad54 sequence variation found in nature (*Top* panel) with the results of the deep mutagenic screen (*Bottom* panel).

We used site directed mutagenesis to assess the importance of the interfacial contacts between Rad51 and Rad54. For this analysis, we used our CryoEM structure to guide the design of *rad54* mutants with various combinations of single or multiple point mutations at amino acid residues shown to make contacts with Rad51 (Fig. 2 *D*–*F*). These *rad54* mutants were cloned in a CEN vector and their functionality was assessed by determining whether cells expressing these mutants could survive when grown on media containing MMS (*SI Appendix*, Fig. S4). Interestingly, most single point mutants within the Rad51 interaction motif of Rad54 had little or no effect on cell survival (*SI Appendix*, Fig. S4*A*). The sole exceptions were L127A, L127D, and F131A. Residue L127 makes hydrophobic contacts with a conserved region of Rad51 (Fig. 2*D*) and residue F131 is part of the FxxP motif and phenylalanine was strongly preferred at this position (Figs. 2*D* and 3 *B* and *C*). Notably, the P134A mutation, which is also part of the FxxP motif, was tolerated in the point mutant assays (see below; *SI Appendix*, Fig. S4).

All *rad54* alleles bearing multiple mutations exhibited genetic defects relative to wild-type *RAD54* (*SI Appendix*, Fig. S4*B*). Four of these contain the L127A mutation, which alone likely accounts for the observed defects (*SI Appendix*, Fig. S4*B*). Additionally, other alleles with multiple mutations in interacting residues yielded significantly reduced growth on MMS-containing plates comparable to the F131A point mutant (*SI Appendix*, Fig. S4 *A* and *B*). Examples of these highly defective mutants include: the *rad54* triple mutant K105D R112D R119D, all of which are located near the N-terminal end of the Rad51 interaction domain (Fig. 2*E*); the *rad54* triple mutant R128D R129D K136D, all located near the C-terminal end of the Rad51 interaction domain (Fig. 2 *E* and *F*), and the *rad54* triple mutant R129A V133A I135A, which are also clustered near the C-terminal end of the Rad51 interaction motif (Fig. 2 *D*–*F*).

### Deep Mutagenic Screen of the Rad51 Interaction Motif from Rad54.

We conducted a deep mutagenic screen to further define the functional significance of the amino acid residues within the Rad51 interaction motif of Rad54. For this assay, we generated libraries of Rad54 mutants in which each amino acid across the entire Rad51 interaction motif was mutated to all other possible amino acid residues (Fig. 3*A*). The *rad54* mutant libraries were encoded within a CEN plasmid (*pRS415–ScRad54*) in which the codons from within the 44 amino acid residue Rad51 interaction motif (spanning Rad54 amino acid residues 101 to 144) were randomized to NNN, where N could be A, G, C, or T, yielding a set of input libraries with 880 different single amino acid mutations, not including mutants in which the codons were changed to stop codons. All functional *rad54* variants were identified based upon their ability to support growth of a *rad54∆* strain on media containing 0.015% MMS (Fig. 3*A*). The resulting pool of survivors were analyzed by next generation DNA sequencing. Fold-enrichment for each variant was calculated by comparing the relative abundance of each allele recovered from cells grown in the absence of MMS versus cells grown in the presence of MMS (Fig. 3 *B* and *C*). All wild-type alleles were enriched in the survivor pool, providing internal positive controls demonstrating that the deep mutagenesis screen could recover the *S. cerevisiae* wild-type *RAD54* alleles (Fig. 3*B*). In contrast, alleles bearing stop codons were depleted, providing internal negative controls confirming that full-length Rad54 was necessary for cell growth on MMS (Fig. 3*B*). Notably, with the exception of the FxxP motif, the functional landscape of the Rad51 interaction motif of Rad54 was largely tolerant of single point mutations, suggesting that no single amino acid residue within the motif was absolutely essential, although there were many instances where particular amino acid residues were not tolerated at some position (Fig. 3 *B*–*D*). For example, in agreement with the point mutation data, the L127A and L127D *rad54* mutants were nine- and 1,679-fold depleted in the deep mutagenic screen compared to wild-type *RAD54*. In addition, the deep mutagenic screen showed that although proline was highly preferred within the FxxP motif, mutations at residue P134 to alanine or glycine were also tolerated, which agreed with the point mutation data (Fig. 3 *B* and *C* and *SI Appendix*, Fig. S4). Last, comparison of the results of the deep mutagenesis to the sequence conservation of the Rad51 interaction motif based upon alignment of Rad54 sequences from 4,836 specifies shows good agreement between the two datasets and affirms that the phenylalanine and proline residues of the FxxP were the among the two most important residues with respect to Rad54 functional interactions with Rad51 (Fig. 3*D*).

### Predicted Structure of Hed1 Bound to Rad51.

**Fig. 4.**
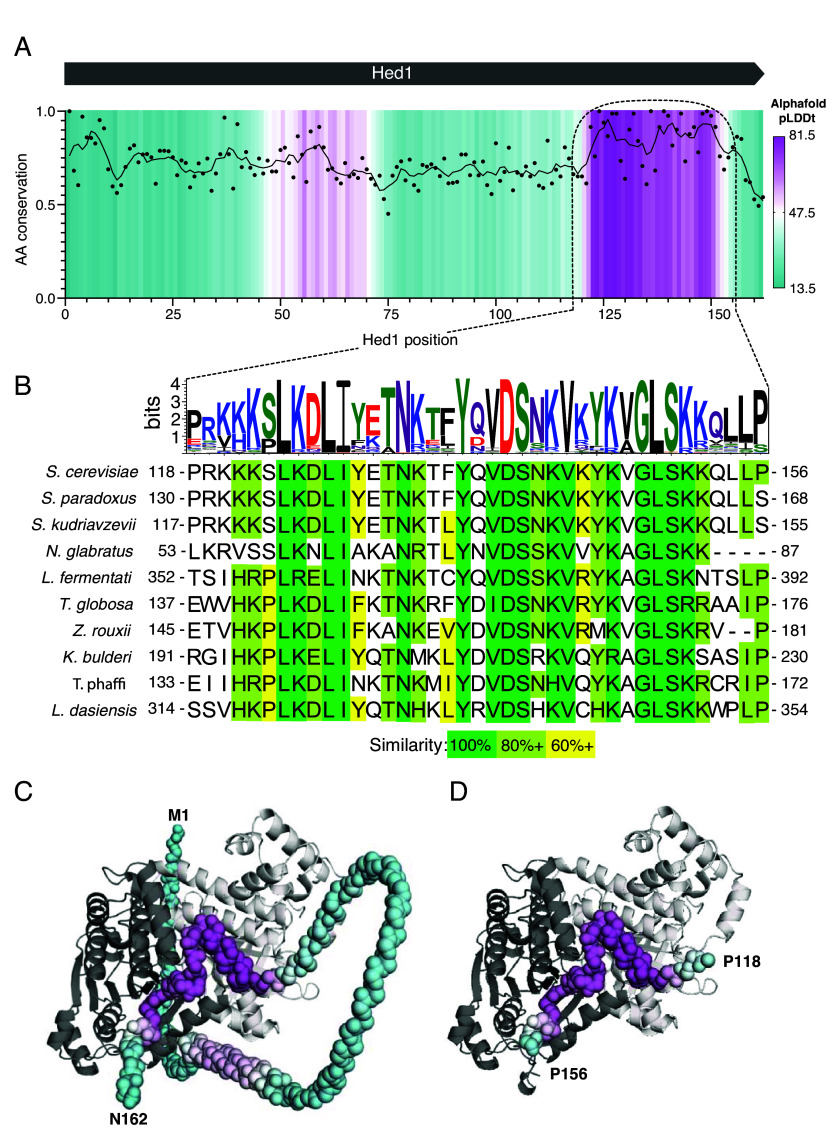
Predicted interaction interface between Rad51 and Hed1. (*A*) Plot of amino acid residue conservation and predicted AlphaFold3 structure (pLDDt; predicted local difference distance test) versus residue number for *S. cerevisiae* Hed1 is shown as in Fig. 1*A*. (*B*) Sequence logo and sequence alignments for the region encompassing the highly conserved C-terminal region of Hed1 that is predicted to fold when bound to Rad51. (*C*) Predicted AlphaFold3 structure for full-length Hed1 (residues M1 to N162) and (*D*) Hed1 residues P118-P156 bound to two adjacent Rad51 monomers from within a Rad51 filament.

Hed1 is a small (162 amino acid residues) meiosis-specific protein found in budding yeast that downregulates Rad51 activity in meiosis by blocking its interactions with Rad54 (52, 53, 55). Based upon our previous single molecule assays, we have proposed that Hed1 acts as a competitive inhibitor of Rad54 binding and, given this model, we predicted that Hed1 would likely interact with the same binding surface on Rad51 as Rad54 (54). As with Rad54, we used bioinformatics and AlphaFold3 predictions to identify potential regions of Hed1 responsible for its interactions with Rad51 (Fig. 4*A*). No known homolog of Hed1 has yet been identified in higher eukaryotes, but Hed1 itself is conserved among the budding yeast and its CTD region contains the most highly conserved portion of the protein (Fig. 4 *A* and *B*). AlphaFold3 modeling of Hed1 alone predicts that the protein is largely unstructured (*SI Appendix*, Fig. S5 *A* and *B*). However, modeling of Hed1 bound to Rad51 suggests that the CTD of Hed1 becomes highly structured upon binding to the Rad51-ssDNA nucleoprotein filament and its predicted binding site also overlapped with that of Rad54, (Fig. 4 *C* and *D* and *SI Appendix*, Fig. S5*C*). Notably, the predicted Rad51 interaction motif from Hed1 does not contain an FxxP motif and its sequence is entirely unrelated to the Rad51 interaction motif from Rad54. Therefore, based upon these findings, we considered the possibility that Hed1 amino acid residues 118 to 156 might represent a unique Rad51 interaction motif distinct from that found in Rad54.

### Structure of the Hed1-Bound Rad51 Presynaptic Complex.

**Fig. 5.**
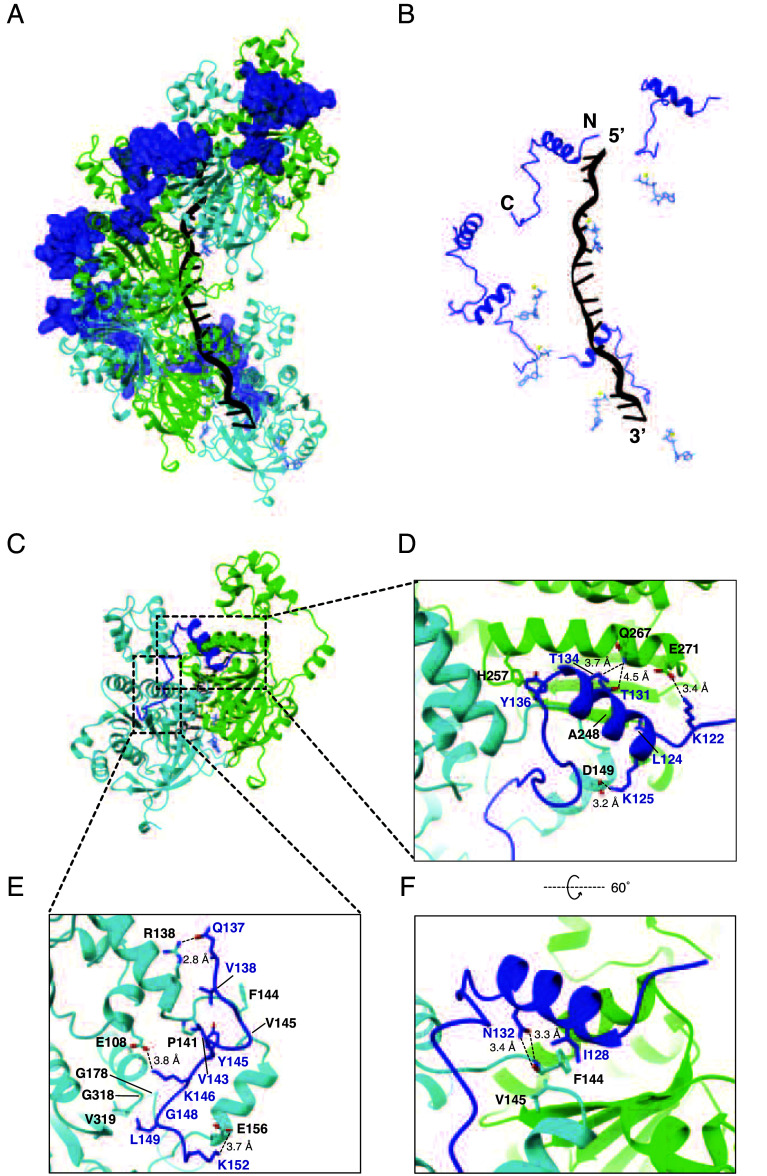
Structure of the Rad51 interaction motif of Hed1 bound to the Rad51-ssDNA filament. (*A*) CryoEM reconstruction of the Rad51-ssDNA filament bound by a peptide encompassing Hed1 residues P118 to P156. Rad51 subunits are shown in alternating colors (cyan and green) and the Hed1 peptide is shown in blue. (*B*) Structure in which the Rad51 proteins have been removed to highlight the relative locations of the Hed1 peptide, Rad51-bound ssDNA, and ATP molecules. (*C*) Structure showing that the Hed1 peptide (blue) is bound to two adjacent Rad51 subunits (the 5′ facing subunit is shown in green and the 3′ facing subunit is shown in cyan). (*D*–*F*) Details of the molecular interactions between Hed1 (blue) and the two adjacent Rad51 subunits.

We obtained a 2.8 Å resolution CryoEM structure of the 39 amino acid residue Hed1 peptide (residues P118 to P156) bound to the Rad51-ssDNA filament and 33 of these residues were visible within the structure (residues K121 to L153; Fig. 5 *A* and *B* and *SI Appendix*, Figs. S6 and S7 and Table S1). Similar to Rad54, the Rad51 binding motif from Hed1 bridges two adjacent Rad51 monomers within the Rad51 filament corresponding to a buried surface area of 1,494.4 Å^2^ (Fig. 5*C*). The ability of Hed1 to interact with two adjacent Rad51 monomers provides a structural explanation for the observation that Hed1 binding stabilizes Rad51 filaments (52). The N-terminal most portion of the Hed1 peptide is composed of a short loop made up of residues P118 to S123, followed by a three-turn alpha helix made up of residues L124 to F135, and it ends with a short loop region composed of residues Y136 to P156 (Fig. 5 *C*–*E*). Most of the Hed1 amino acid residue contacts from this region involve a single alpha helix within the Rad51 protomer facing the 5′ end of the ssDNA (Fig. 5*D*). These include Hed1 residues K122, T131, T134, and Y136 which are within hydrogen bonding distance of Rad51 residues E271, Q267, and H257, and Hed1 residue L124 which is involved in hydrophobic interaction with Rad51 residue A248 (Fig. 5*D*). Three additional Hed1 residues within this region contact the second Rad51 protomer: K125 makes contacts with Rad51 residue D149; residue I128 is involved in hydrophobic interactions with Rad51 residues F144 and V145; and residue N132 which makes hydrogen bond contacts with the backbone amino and carboxyl atoms of Rad51 residue F144 (Fig. 5*F*). The C-terminal most portion of the Hed1 peptide is composed of an extended loop-like region that interacts with the second Rad51 protomer via electrostatic and hydrophobic contacts (Fig. 5*E*). The electrostatic contacts include Hed1 residues Q137, K146, and K152, which contact Rad51 residues R138, E108, and E156, respectively (Fig. 5*E*). Hydrophobic contacts include Hed1 residues V138, V143, Y145, and L149, which interact with a hydrophobic pocket present on the surface of Rad51 composed of residues A137, V140, P141, M142, G143, F144, G318, V319, and A320 (Fig. 5*E*).

Four Hed1 mutations have previously been identified that disrupt its interactions with Rad51, which include I128M, T131P, N132S, and N132I (52). Our structure shows that I128 is involved in a hydrophobic interaction Rad51 residues F144 and V145, and the longer methionine side chain would likely induce a steric clash with these residues; and the Hed1 T131P mutation would abolish a hydrogen bonding interaction with Rad51 residue Q267 and disrupt helical secondary structure. The N132S and N132I mutations likely disrupt interactions with the backbone amino and carboxyl atoms of Rad51 residue F144. Another Hed1 mutant, Hed1 Δ114 to 122, can bind to Rad51 but its binding interaction is weakened sufficiently that it can no longer block the binding of Rad54 (52, 54). Residues 114 to 117 lie just outside of the region that we define as the Rad51 interaction motif of Hed1, residues 118 to 120 are not visible in the structure, and residue 121 does not appear to interact with Rad51. However, residue K122 interacts with Rad51 residue E271, and disruption of this contact may contribute to the weakened binding observed for the Hed1 Δ114 to 122.

Most notably, the Hed1 peptide binds across the same surface of Rad51 as does Rad54, albeit through an entirely different mechanism, and it is in fact bound in the complete opposite orientation to that observed for Rad54 (c.f. Figs. 2 and 5). The Hed1 and Rad54 binding sites overlap with one another, such that the binding of Hed1 and Rad54 would be mutually exclusive, providing a clear structural explanation for prior single molecule studies showing that Hed1 acts as a competitive inhibitor of Rad54 binding (54).

### Genetic Analysis of the Rad51 Interaction Motif from Hed1.

To assess the biological relevance of the Hed1 contacts observed in the CryoEM structure, we adapted an assay in which the expression of Hed1 during mitosis causes cells to become sensitized to Rad51-mediated repair of MMS-induced DNA damage (55). As expected, expression of wild-type Hed1 in mitotic cells resulted in sensitivity to MMS, whereas the empty vector control did not (*SI Appendix*, Fig. S8*A*). We also tested the previously published *hed1* mutants I128M, T131P, and N132S, all of which allowed for cell growth on MMS (*SI Appendix*, Fig. S8*A*), consistent with previously published results (52). In striking contrast to Rad54, single alanine point mutations in most residues that make specific contacts between Hed1 and Rad51 resulted in the loss of Hed1-mediated inhibition of cell growth, including residues K122, L124 K125, T131, Y136, V138, V143, K146, L149, and K152 (*SI Appendix*, Fig. S8*A*). The exceptions were T134A, which exhibited a phenotype comparable to wild-type Hed1, and Q137A, which yielded a mild phenotype consistent with partial inactivation of Hed1 activity (*SI Appendix*, Fig. S8*A*). Notably, all *hed1* variants tested that contained multiple mutations exhibited null phenotypes (*SI Appendix*, Fig. S8*B*). Together, these genetic assays provide support for the biological relevance of the protein–protein interface observed on our CryoEM structure of the Rad51-Hed1 complex.

## Discussion

Here we have identified the Rad51 interaction motif within Rad54, and we have described the structural basis for Rad54 interactions with the Rad51 presynaptic complex. We have also provided the structural basis for how the interaction between Rad51 and Rad54 is negatively regulated by the meiosis-specific protein Hed1 allowing for meiotic recombination to be driven by meiosis-specific recombinase Dmc1. Together, our findings provide atomic-level insights into the spatial and temporal interplay between Rad54, Hed1, Rad51, and Dmc1 that takes place during the transition from mitotic to meiotic recombination in *S. cerevisiae*.

### Rad54 Interactions with the Rad51 Presynaptic Complex.

The Rad54 binding sites on Rad51 span the length of the presynaptic complexes suggesting that Rad54 could, in principle, act in approximately 1:1 stoichiometry with Rad51 (Fig. 2). This finding is consistent with previous in vitro biochemical studies which suggested an optimal ratio of one molecule of Rad54 for each molecule of Rad51 during strand invasion and 1:1 stoichiometry observed in saturated pulldown experiments (28, 31). However, the stoichiometric details of in vivo Rad54-Rad51 complexes remain unclear, and prior single molecule studies show that the homology search can be supported by substoichiometric levels of Rad54 (30). These considerations raise the question of whether Rad51 in cells might be bound by Rad54 monomers at locations distributed randomly along the length of the presynaptic filament, or whether Rad54 might form localized clusters of more well-defined higher-order complexes.

Notably, the Rad54 binding pocket spans two adjacent Rad51 monomers within the Rad51 presynaptic complex (Fig. 2*C*), which can explain two important influences of Rad54 on Rad51 behavior. First, the ability of Rad54 to bridge two adjacent Rad51 monomers within the filament provides a plausible explanation for how Rad54 stabilizes the presynaptic complex (32, 64). Thus, Rad54 may act as a molecular “staple” that helps maintain nucleoprotein stability. Second, the existence of a composite binding pocket composed of two adjacent Rad51 monomers provides a straightforward structural explanation for why Rad54 is recruited to sites of DNA damage only after the initial recruitment of Rad51 (65), in that the composite Rad54 binding pocket would only exist after the assembly of Rad51 filaments onto the ssDNA overhangs present at processed DSB. Thus, the existence of a composite binding surface for Rad54 on the surface of the presynaptic complex likely serves as a regulatory mechanism for controlling the timing of Rad54 recruitment to the sites of DNA damage.

Besides mediating interactions with Rad51, the Rad54 NTD has also been shown to contain a DNA-binding determinant that regulates branched DNA substrate preference (35, 39). As the Rad51-binding domain identified here includes only 34 residues of the entire ~260 amino acid Rad54 NTD from budding yeast (smaller in most organisms, e.g., ~155 amino acids in human Rad54), ample protein sequence remains to directly interact with DNA as well as bridge these modules with flexible peptide linkers to conform to spatial requirements of the active Rad54-Rad51 complex. Furthermore, the Rad54 NTD is largely nonconserved in diverse species, with the exception of the Rad51-interaction motif described here, and residues that appear to anchor the NTD to the CTD core (Fig. 1, the latter includes residues ~150 to 180), presenting an opportunity for clade-specific adaptations in different organisms, e.g., possible expanded functions of the extended NTD in fungi.

### Conservation of the Rad51-Rad54 Binding Mechanism.

**Fig. 6.**
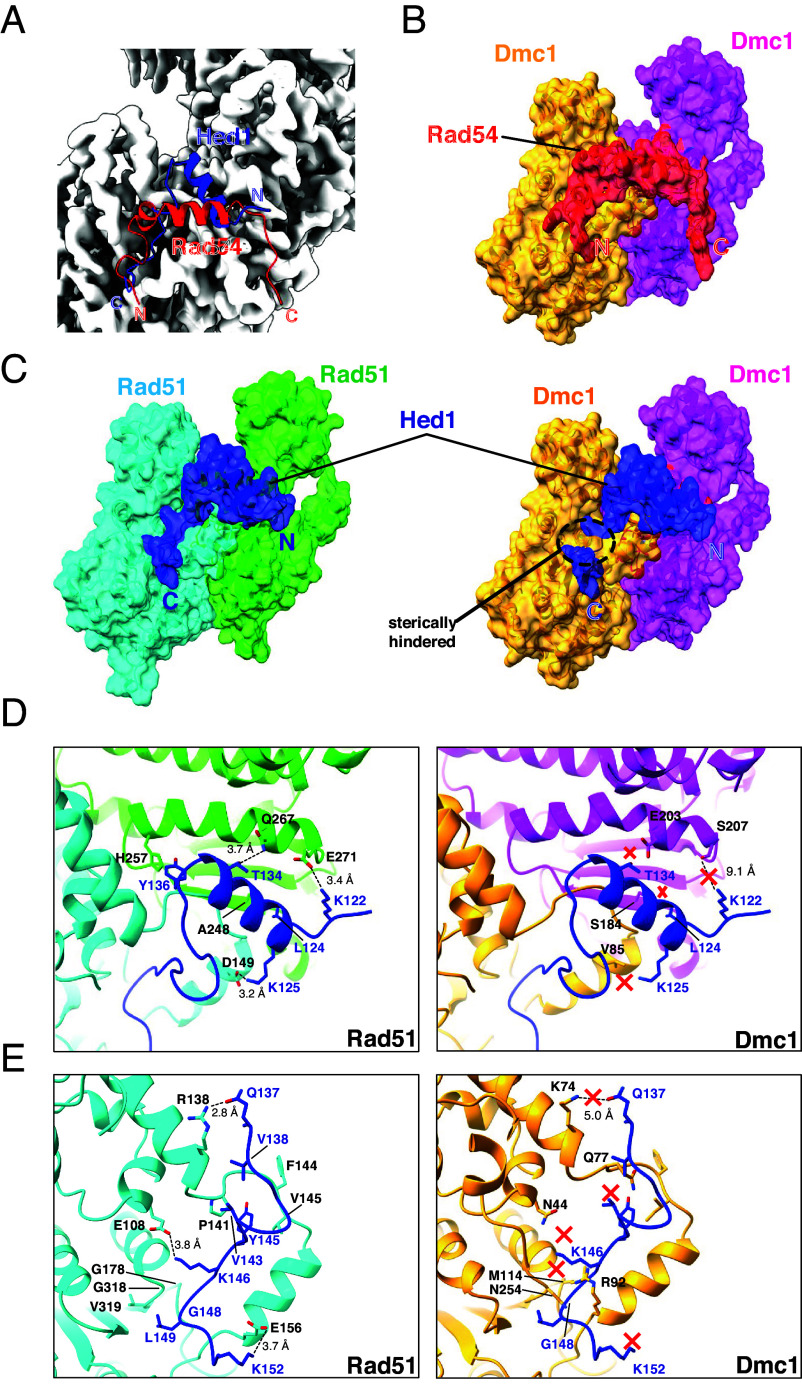
Structural basis for the mechanisms of Rad51 regulation in meiosis. (*A*) Model showing that the Rad51 interaction motifs from Rad54 (red) and Hed1 (blue) overlap with one another when bound to Rad51 (gray) but are oriented in the opposite directions relative to their N and C termini. (*B*) Illustration showing that the Rad51 interaction motif peptide from Rad54 (red) can be docked onto the structure of *S. cerevisiae* Dmc1 (adjacent subunits shown in orange and magenta). (*C*) Side-by-side comparison of the Rad51 interaction motif from Hed1 (blue) bound to either Rad51 (adjacent subunits shown in cyan and green) or docked onto Dmc1 (adjacent subunits shown in orange and magenta). (*D* and *E*) Side-by-side comparisons of the molecular contacts between Hed1 and Rad51 or Hed1 and Dmc1. Hed1 contacts that are predicted to be disrupted with Dmc1 are highlighted with a red “x.”

Our CryoEM data show that contacts with Rad51 span a region within *S. cerevisiae* Rad54 encompassed by amino acid residues R103 to K136. Interestingly, with the exception of the phenylalanine and proline residues within the FxxP motif, our deep mutagenic analysis reveals that the functional landscape of the Rad51 interaction motif of Rad54 is highly tolerant to single amino acid variants, which is also in good agreement with the sequence conservation data (Fig. 3). Importantly, the essential phenylalanine and proline of the Rad54 FxxP sequence motif is conserved from yeast to humans as is the corresponding binding pocket on the Rad51 filament (*SI Appendix*, Fig. S9), suggesting that the mode of interaction defined by our CryoEM structure of the Rad54 peptide bound to the Rad51 presynaptic complex may be broadly conserved. Consistent with this conclusion, AlphaFold3 predictions for human RAD54 provide further evidence for a conserved mode of interaction, with a similar predicted backbone fold and hydrophobic contacts (*SI Appendix*, Fig. S9 *A*–*C*). This hypothesis is further supported by docking of the Rad54 peptide into our previous structure of *S. cerevisiae* Dmc1 [PDB: 9D4N; (66)], which reveals that all of the major contacts are maintained (Fig. 6*B*). In addition, the FxxP binding pocket is also conserved in both yeast and human Dmc1 (*SI Appendix*, Fig. S9 *C* and *D*), strongly suggesting that the same mode of interaction may be utilized for Rad54 interactions with Dmc1 (see below).

Interestingly, human RAD51 binds to the tumor suppressor protein BRCA2 via the RAD51-binding TR2 motif located within the C-terminus of BRCA2 (67–72). This interaction also involves an FxxP motif found within BRCA2 (67, 68) and the binding site for the TR2 motif overlaps with the same site where we predict that human RAD54 will interact with RAD51 (*SI Appendix*, Fig. S9*E*). Thus, accumulating evidence suggests that multiple factors may interact with same binding cleft on the surface of the Rad51-ssDNA presynaptic complex, which implies the possible requirement for different regulatory mechanisms to control the spatial distribution and temporal association of required factors at various stages of the HR, such as Rad51 filament assembly, the homology search, strand invasion, and Rad51 filament disassembly.

Rdh54 is a closely related homolog of Rad54, and it also participates in HR (73–75). Rdh54 stimulates D-loop formation in vitro (76, 77), removes Rad51 and Dmc1 from dsDNA (22, 78, 79), and acts together with Rad54 to enhance Rad51 presynaptic filament stability (32), promotes template switching during break-induced replication (BIR) (80), and plays a greater role in interhomolog recombination compared to Rad54 (74, 75, 78). Notably, Rdh54 and Rad54 both bind to the Rad51 presynaptic complex through their disordered NTDs, but they interact through different binding mechanisms (58). The evidence supporting this idea is that i) Rad54 and Rdh54 can both simultaneously interact with the presynaptic complex without interfering with one another, ii) Hed1 inhibits Rad54 binding to Rad51 but has no effect on Rdh54 binding, but iii) Hed1 can be made to inhibit Rdh54 just by swapping the NTDs between Rad54 and Rdh54 (58). Therefore, we predict that Rdh54 may display a mode of interaction with the Rad51-ssDNA presynaptic complex that will be distinct from that of both Rad54 and Hed1.

### Structural Basis for the Regulatory Control of Rad51 and Dmc1 in Meiosis.

Rad51 is the only recombinase that is expressed in mitosis and during HR-dependent DNA repair it drives strand invasion primarily between sister chromatids. Entry into meiosis coincides with the expression of the meiosis-specific recombinase Dmc1 (81) and the meiosis-specific protein Hed1, which downregulates Rad51 strand exchange activity (55). As a consequence, Dmc1 is considered to be the active recombinase responsible for strand exchange between homologous chromosomes in meiosis (41, 44). Our data provide a structural explanation for how this regulatory transition takes place in budding yeast. Our CryoEM structures show that Hed1 and Rad54 bind to the same surface cleft on Rad51 such that their binding interactions would be mutually exclusive (Fig. 6*A*), and we have previously shown that these binding interactions are so tight that they show no evidence for reversible dissociation in vitro on time scales that might be considered biologically relevant (54).

In contrast to Rad54, which interacts with both Rad51 and Dmc1, Hed1 is highly specific for just Rad51 and does not bind to Dmc1 (54). This binding specificity ensures that Hed1 can selectively downregulate Rad51 strand exchange activity in meiosis without affecting the strand exchange activity of Dmc1. Our structural studies provide a plausible explanation for this specificity by showing that there would be extensive steric clashes between the C-terminal region of the Hed1 peptide and Dmc1 if the same binding mode was preserved (Fig. 6*C*). There are also numerous other Rad51 residues that make contacts with Hed1 that are different in Dmc1 such that they disrupt essential protein–protein contacts (Fig. 6 *D* and *E*). For example, Rad51 residues E108, E156, D149, and Q267 are instead N44, R92, V85, and E203 in Dmc1, all of which result in the disruption of specific residue interactions (Fig. 6 *D* and *E*). Rad51 residue R138 is K74 in Dmc1 and would be positioned 2 Å further away from Hed1 residue Q137, perhaps weakening this interaction. Rad51 residue P141 aligns with residue Q77 in Dmc1, resulting in the loss of a hydrophobic interaction (Fig. 6*E*). Rad51 residue E271, which interacts with Hed1 residue K122, is equivalent to S207 in Dmc1 and is positioned too far away to form a hydrogen bonding interaction (Fig. 6*D*). Last, Rad51 residues G178 and G318 are instead M114 and N254 in Dmc1, which would sterically clash with Hed1 (Fig. 6*E*).

### Regulation of Rad54 Binding Via Posttranslational Modification.

In *S. cerevisiae*, an additional level of regulatory control is exerted over Rad54 during meiosis via Mek1-dependent phosphorylation of T132 (51), which may weaken interactions with Rad51 by adding a phosphate group immediately adjacent to the Rad54 FxxP motif that is in close proximity to the PAP of Rad51 (Fig. 2*F*). Although Rad54–Rad51 interaction mechanism may be broadly conserved (*SI Appendix*, Fig. S9), its regulation through Mek1-dependent phosphorylation of residue T132 may be specific to budding yeast given that threonine 132 is not conserved and is instead replaced with lysine in many higher eukaryotes (Fig. 1*C*). Hed1 is a substrate for Mek1-dependent phosphorylation as well at T40, enhancing protein stability to maintain a sufficient effective concentration, thus indicating a coordinated phosphorylation control of Rad51 interaction partners during meiosis (82).

## Methods

### CryoEM Sample Preparation.

Peptides sequences were as follows: Rad54 peptide, 44 amino acids, derived from residues 101 to 144 of *S. cerevisiae* Rad54: 101-T K R R K D A L S A Q R L A K D P T R L S H I Q Y T L R R S F T V P I K G Y V Q R H S L-144; and the Hed1 peptide, 39 amino acids, derived from residues 118 to 156 of *S. cerevisiae* Hed1: 118-P R K K K S L K D L I Y E T N K T F Y Q V D S N K V K Y K V G L S K K Q L L P-156. Peptides were synthesized by Genscript (>95% purity) and were resuspended in phosphate buffered saline (PBS) + 1 mM DTT to a final concentration of 400 µM for the Rad54 peptide or 200 µM for the Hed1 peptide.

To assemble the nucleoprotein filaments, Rad51 (5 μM) and a 96-mer ssDNA (0.25 μM) were preincubated for 10 min at 30 °C in HR buffer (30 mM Tris [pH 7.5], 20 mM MgCl_2_, 50 mM KCl, 1 mM DTT, 2 mM ATP). To form the Rad51-Rad54 peptide and Rad51-Hed1 peptide complexes, an eightfold molar excess (40 μM) of the either the Rad54 peptide or the Hed1-peptide was added to the preassembled Rad51-ssDNA nucleoprotein filaments and reactions were further incubated for an additional 10 min at 30 °C. A 3.5 μL aliquot of these samples was applied to glow-discharged UltrAuFoil R 0.6/1 Au300 grid for the Rad54 peptide and AuFlat 1.2/1.3 grid for Hed1 peptide, blotted for 5 s with a force 3, and plunge-frozen in liquid ethane using a Vitrobot Mark IV (FEI, USA) with 100% humidity at 4 °C.

### Cryo-EM Data Acquisition.

Samples were initially screened using a Glacios (Thermo Fisher, 200 keV) at Columbia University Irving Medical Center. Grids selected for high-resolution data collection were imaged using a Titan Krios microscope (Thermo Fisher) operated at 300 keV and equipped with a K3 direct electron detector at the New York Structural Biology Center. For the Rad54 complex and the Hed1 complex, the magnification was 105 K (pixel size = 0.844 Å/pixel) and 85 K (pixel size = 1.083 Å/pixel) collected in electron counting mode, with a total dose of 59.51 e^−^/Å^2^ and 51.21 e^−^/Å^2^, respectively. Defocus range was −0.8 to −2.5 for both datasets.

### CryoEM Data Processing.

Data were processed using CryoSPARC v4.3.1 (83). For the Rad54 peptide complex, a total of 8,367 movies were collected and aligned using patch motion correction and patch CTF estimation (84). After manual inspection of micrographs for quality such as ice contamination, 246 micrographs were discarded, and 8,121 micrographs were used for downstream processing. Initially, 995,541 particles were picked and extracted with a box size of 256×256 followed by iterative 2D classification to remove junk/denatured and nonhelical particles. The selected 434,806 particles were used for generating 2 ab initio 3D density maps to generate an initial filament volume and clean out residual junk particles. 363,379 particles were used for the reconstruction and refinement of the final 3D map. The nominal resolution of the density map of 3.26 Å was estimated by 0.143 gold standard Fourier shell correlation (FSC) cut off (*SI Appendix*, Fig. S2).

For the Hed1 peptide complex, a total of 6,212 micrographs were collected and aligned using patch motion correction and patch CTF estimation. After manual inspection of micrographs for quality, 758 micrographs were discarded, and 5,454 micrographs were used for downstream processing. Initially, 1,184,183 particles were picked and extracted with a box size 256×256 followed by iterative 2D classification to remove junk/denatured and nonhelical particles. The selected 895,334 particles were used for generating 2 ab initio 3D density maps to generate an initial filament volume and clean out residual junk particles. 876,496 particles were used for the reconstruction and refinement of the final 3D map. The nominal resolution of the density map of 2.81 Å was estimated by 0.143 gold standard FSC cut off (*SI Appendix*, Fig. S6).

### Structure Refinement and Analysis.

Our previous structure of the Rad51-ssDNA filament [PDB: 9D46; (66)] was used as a guide in building the atomic models. The Alphafold3 predicted peptide structures were used as a reference in manual fitting of the structures into the cryo-EM density map using Chimera (85). After initial rigid-body refinement in Phenix (86), amino acid residue sidechains were manually inspected and corrected for fitting into the density map using Coot (87). Fitted models were real space refined with the secondary structure and Ramachandran restraints in Phenix (86).

Surface Area was calculated using PDBe PISA v1.52 (https://www.ebi.ac.uk/pdbe/prot_int/pistart.html) (88). The Rad51 dimer was considered as one structural unit and either the Rad54 or Hed1 peptide was considered as another structural unit for the calculation. All potential electrostatic interactions shown in the figures were verified using the FindHBond tool in UCSF Chimera with the geometric distance and angle constraints set to <4 Å and 110 to 180° (85).

### Additional Methods.

Details of the Bioinformatic analysis, protein purification, AlphaFold3 structure predictions, biochemical assays, and genetic assays are provided as an *SI Appendix*.

## Supplementary Material

Appendix 01 (PDF)

Dataset S01 (XLSX)

Dataset S02 (XLSX)

Dataset S03 (XLSX)

## Data Availability

All oligonucleotide sequences used in this study are provided in Dataset S1, the deep mutagenesis data are provided in Dataset S2, and yeast strain information is provided in Dataset S3. The CryoEM structures of Rad54-Rad51-ssDNA and Hed1-Rad51-ssDNA have been deposited in the Research Collaboratory for Structural Bioinformatics (RSCB) Protein Data Bank (PDB) with accession codes: 9E6L (89) and 9E6N (90). The CryoEM density maps of Rad54-Rad51-ssDNA and Hed1-Rad51-ssDNA have also been deposited in the Electron Microscopy Data Bank (EMDB) with accession codes: EMD-47572 (91) and EMD-47573 (92). All original code has been deposited in Github and is publicly available at the following URL: https://github.com/michaeltpetassi/Rad54Hed1_2025 (93). Deep sequencing data have been deposited at the NCBI Sequence Read Archive (SRA) and are publicly available via the BioProject ID: PRJNA1235687 (94). All accession codes and data access information are also summarized in *SI Appendix*, Table S2.
