# Supplementary material for: Structural basis for Rad54- and Hed1-mediated regulation of Rad51 during the transition from mitotic to meiotic recombination
Source: Proc Natl Acad Sci U S A. 2025 Sep 11;122(37):e2510007122. doi: 10.1073/pnas.2510007122 (PMC12452912; doi:10.1073/pnas.2510007122)
Supplement: Supplementary file 1 — Appendix 01 (PDF) [file pnas.2510007122.sapp.pdf]

SI Appendix for:

Structural basis for Rad54- and Hed1-mediated regulation of Rad51 during the transition from mitotic to meiotic recombination

Yeonoh Shin[1*], Michael T. Petassi[1*], Aidan M. Jessop[1], Stefan Y. Kim[1], Razvan Matei[1†], Katherine Morse[1], Vivek B. Raina[1], Upasana Roy[1‡], and Eric C. Greene[1]

[1]Department of Biochemistry & Molecular Biophysics, Columbia University Irving Medical Center, New York, NY, 10032, USA
*Equal contribution

†Present address: NYU Grossman School of Medicine, Research Building 3-530P, 540 1st Ave, New York, NY 10016

‡Present address: HHMI Janelia Research Campus, 19700 Helix Dr, Ashburn, VA 20147

To whom correspondence should be addressed: ecg2108@cumc.columbia.edu

This PDF file includes:

Supporting Methods text
Figures S1 to S9
Table S1 to S2
SI References

Other supporting materials for this manuscript include the following:

Dataset 1 to Dataset 3

SUPPORTING METHODS

Bioinformatics

For Rad54, *S. cerevisiae* S288C RAD54 (NP_011352) and *S. cerevisiae* S288C Rdh54 (NP_011106) were used as queries for blastp searches with an E-value threshold of 1e-60 against the NCBI nr protein database accessed in July 2023 (1). Sequences shorter than 650 amino acids or longer than 1300 amino acids were discarded, resulting in 10,401 unique sequences. Sequences were clustered to 60% identity using cd-hit (2) resulting in 993 clusters, representatives aligned with MUSCLE (3), and a similarity tree was built using Genieous Prime 2023.1 https://www.geneious.com). Sequences clustered into three clades, corresponding to Rad54, Rdh54, and a small group of distantly related helicases XRCC6. The Rad54 clade (4,855 sequences) was extracted, clustered to 80% identity using cd-hit (898 clusters) and aligned with MUSCLE. For Hed1, *S. cerevisiae* S288C HED1 (NP_001035220) was used as query for blastp search with an E-value threshold of 0.05 against the NCBI nr protein database accessed in June 2024 (1). The 87 resulting sequences were aligned with MUSCLE.

Protein purification

S. cerevisiae Rad51 was overexpressed in *E. coli* BL21 (DE3) Rosetta2 cells transformed with a plasmid encoding 6XHis-SUMO-ScRad51. Cells were grown in 2L LB media containing 100 µg/ml carbenicillin and 35 µg/ml chloramphenicol at 37°C until OD600 = 0.6, induced with 0.5 mM IPTG and grown for 3 hours at 37°C. The cell paste was suspended in 50 ml lysis buffer (50 mM Tris-HCl [pH 7.5], 10% glycerol, 1M NaCl, 1mM DTT, 0.5 mM PMSF, 15 mM imidazole, 0.1% tween 80, 1 protease inhibitor tablet) and lysed by sonication. The lysate was centrifuged at 35,000 rpm for 45 mins followed by precipitating the supernatant with 12g ammonium sulfate for 1 hour. The precipitate was spun down at 10,000 rpm for 30 mins. The pellet was dissolved in 50 ml binding buffer (25 mM Tris-HCl [pH 7.5], 10% glycerol, 200 mM NaCl, 0.1% Triton X-100, 15 mM imidazole, 5 mM beta-mercaptoethanol) and applied to the 5 ml HisPur™ Ni-NTA resin (Thermo Fisher Scientific) equilibrated with the same binding buffer. The protein was eluted with 10 ml elution buffer (25 mM Tris-HCl [pH 7.5], 10% glycerol, 200 mM NaCl, 200 mM imidazole, 0.1% Triton X-100). SUMO protease was added to the elution, followed by dialyzing for 16 hours at 4˚C in dialysis buffer (50 mM Tris-HCl [pH 7.5], 200 mM NaCl, 10% glycerol, 15 mM imidazole, 1 mM DTT). The sample was re-applied to the 5 ml Ni-NTA resin equilibrated with the binding buffer and flow-through was collected and concentrated to 30 µM. The protein was flash-frozen in liquid nitrogen and stored at –80˚C.

The Rad51 triple mutant protein (D239A, D241A, D242A) was purified in the same way as wild-type Rad51, except it was dialyzed and stored in buffer with 500 mM NaCl to prevent aggregation. In brief, 6xHis–SUMO–Rad51-D239A, D241A, D242A was overexpressed in *E. coli* BL21 (DE3) Rosetta2 cells at 37˚C to an OD_{600} of 0.4–0.6. Expression was induced by addition of 0.5 mM IPTG for 3 h at 37°C. Cells were harvested and stored at –80˚C. Cells were lysed in Cell Lysis Buffer (30 mM Tris–HCl [pH 8.0], 1 M NaCl, 10% glycerol, 10 mM imidazole, 1 mM DTT, 200 mM PMSF, 10% NP-40 and protease inhibitor cocktail (Roche, Cat. No. 05892953001)) by sonication for 10 (s) on and 30 (s) off for a total time of 2 (min). The lysate was clarified by ultracentrifugation at 100,000 x g for 45 min at 4˚C and proteins precipitated with 40% ammonium sulfate. The precipitated proteins were redissolved in Cell Lysis Buffer and bound to 1.5 mL of pre–

equilibrated Ni–NTA resin. The resin was then washed 3X with CLB and eluted in CLB + 200 mM imidazole. The eluted fraction was mixed with 400 units of the SUMO protease Ulp1 (Sigma–Aldrich, Cat. No. SAE0067–2500UN) and dialyzed overnight at 4˚C into Rad51 buffer (30 mM Tris–HCl [pH 8.0], 500 mM NaCl, 1 mM EDTA, 10% Glycerol, 10 mM imidazole). The 6xHis–SUMO tag and SUMO protease were removed by passing the dialyzed proteins over a second 1.5 mL Ni–NTA column. The purified Rad51-D239A, D241A, D242A protein was stored at –80˚C in single use aliquots.

For use in D-loop assays, full-length Rad54 was purified as previously described (4). A protease deficient yeast strain was transformed with GST–tagged Rad54 on a 2–micron plasmid under the control of the Gal1 promoter. Cells were grown in Yeast Nitrogen base (–URA) plus 3% Glycerol and 2% lactic acid. When the cells reached an OD_{600} of 1.5, expression was induced by the addition of 2% galactose for 6 hours. Cells were harvested and stored at –80˚C. Cell pellets were re-suspended in Rad54 re-suspension buffer (30 mM Tris–HCl [pH 7.5], 1 M NaCl, 1 mM EDTA, 10% glycerol, 10 mM BME (β-mercaptoethanol), Protease inhibitor cocktail (Roche Cat. No. 05892953001) and 2 mM PMSF. Cells were disrupted by manual bead beating, and the lysate was clarified by ultracentrifugation at 100,000xg for 1 hour. The lysate was fractionated by ammonium sulfate (AS) precipitation. AS was gradually added with mixing to a final concentration of 20% followed by centrifugation at 10,000Xg for 10 minutes. The supernatant was discarded, and the AS concentration was raised to 50% followed by centrifugation at 10,000x g for 10 min. The protein pellet was re–suspended in PBS (phosphate buffered saline) plus 1 M NaCl and 10 mM BME. The resulting re–suspended protein was then bound to pre–equilibrated GST resin in batch for 1 hour at 4˚C. The GST resin was washed with PBS plus 1 M NaCl and washed again with PBS plus 500 mM NaCl. The protein was then eluted with 20 mM glutathione in PBS plus 500 mM NaCl. The peak fractions were pooled and the applied to a Sephacryl S–300 High Resolution gel filtration column (GE Healthcare, Cat. No. 17–0599–10) pre–equilibrated with Rad54 SEC buffer (30 mM Tris-HCl [pH 7.5], 500 mM NaCl, 1 mM EDTA, 10 % glycerol, and 10 mM BME. The protein eluted in two peaks, one peak occurred outside of the exclusion volume of the column and was discarded. The second peak eluted near the expected MW of a Rad54 monomer and was collected. The monomeric Rad54 peak was pooled and dialyzed against Rad54 SEC buffer plus 50 % glycerol and stored in at –80°C in single use aliquots.

AlphaFold3 structure predictions

Alphafold3 predictions were performed using the Google DeepMind Alphafold3 Server (https://golgi.sandbox.google.com/)(5) with the following molecules - recombinase (*S. cerevisiae* Rad51 UniProt P25454, *H. sapiens* Rad51 UniProt Q06609, *S. cerevisiae* Dmc1 UniProt P25453, or *H. sapiens* Dmc1 UniProt Q14565), poly(dT) ssDNA, ATP, Mg2+, and one interacting protein (*S. cerevisiae* Rad54 UniProt P32863, *S. cerevisiae* Hed1 UniProt Q03937, *H. sapiens* RAD54L UniProt Q92698).

Yeast transformations

Banked frozen yeast strains were streaked onto solid YPD media (1% yeast extract (Sigma-Aldrich, Cat No. 92144), 2% bacto-peptone (Sigma-Aldrich, Cat. No. 91249), 2% glucose (Sigma-Aldrich, Cat. No. G8270), 2% agar (Sigma-Aldrich, Cat. No. G8270) and grown for 2-3 days at 30˚C (all strain information is presented in Dataset 3). Cultures inoculated from single colonies were grown overnight in liquid YPD media and diluted 1:50 into fresh YPD to approximate OD_{600} = 0.4. After 4-hour incubation at 30˚C, cells were harvested by centrifugation at 3,000 rcf (Relative Centrifugal Force) for one minute,

washed three times in at least 1/40 culture volume 0.1 M lithium acetate (Sigma-Aldrich, Cat. No. 517992) and resuspended in a final 1/125 culture volume 0.1 M lithium acetate. 50 μL competent cells were added to 360 μL transformation mix containing 33.33% PEG-3350 (Sigma-Aldrich, Cat. No. 202444), 0.1 M lithium acetate, 10 μg sheared salmon sperm DNA (Thermo Scientific, Cat. No. AM9680, incubated at 95˚C for 5 minutes then kept on ice until use), and up to 2 μg transforming plasmid. Mixes were incubated at 30˚C for 30 minutes with shaking, then incubated at 42˚C for 20 minutes without shaking. Cells were harvested at 3,000 rcf for 90 seconds, supernatant removed and resuspended in 100 μL sterile water before plating on synthetic dropout (SD) media (0.171% YNB (Sunrise Science Products, Cat. No. 1500), CSM lacking tryptophan (-Trp), leucine (-Leu) or uracil (-Ura) (Sunrise Science Products, Cat. No. 1007/1005/1004, recommended amount), 0.5% ammonium sulfate (Sigma-Aldrich, Cat. No. 517992), 2% glucose, 2% agar). Reactions were scaled up by 20-times in 50 mL conical tubes, as needed.

Rad54 deep mutagenesis screen

RAD54 and 500-bp upstream sequence was cloned into pRS415 (centromeric origin, *LEU2* marker) by generating three fragments by PCR amplification (PrimeSTAR Max DNA Polymerase, Takara Bio, Cat. No. R045B): two from *RAD54 S. cerevisiae* W303 genomic DNA covering +500-*RAD54* with an overlap region producing a silent mutation at K152 (A456G) to disrupt a 10-adenine tract with oligonucleotide primers MTP85/MTP135 and MTP86/MTP134; and a third PCR fragment from purified pRS415 with oligonucleotide primers VBR15/VBR16 (all oligonucleotide sequences are presented in Dataset 1). PCR products were digested with DpnI (20 units; NEB, Cat. No. R0176), assembled using In-Fusion Snap Assembly (Takara Bio, Cat. No. 638947) and transformed into Stellar competent *E. coli* cells (Takara Bio, Cat. No. 636763).

Mutant libraries of *pRS415-rad54* for deep mutagenesis screens were generated by single-fragment amplification of *pRS415-RAD54* with forward primer containing 9-bp mixed-base sequence at each 3-codon window and reverse primer generating 15-bp proximal sequence overlap for assembly protocol as above. A minimum of one million transformants were collected in LB supplemented with 100 µg/mL carbenicillin, diluted 1:20 in the same media (to OD_{600} of 2-3), and incubated at 37˚C with shaking for four hours before plasmids were extracted. For pools randomized at position P117, the P117M allele was consistently absent from all mutant pools using randomized top-strand annealing primers; cloning with alternate randomized bottom-strand annealing primers did produce the P117M allele. For the P117 position, this position was randomized individually (i.e. 3-bp mixed-base sequence not 9-bp mixed-base sequence) as performed for all other mutant pools, and read analysis was adjusted accordingly.

The *pRS415-rad54* mutant libraries were transformed into *S. cerevisiae* strain yECG46 as described above. After a 72-hour incubation at 30˚C, ≥1,000,000 transformants were collected, resuspended to a calculated OD = 100, and 100 μL were plated on SD –Leu supplemented with 0.015% MMS. After a 72-hour incubation at 30˚C, ≥10,000 colonies were collected. Plasmids were extracted from cell populations before and after MMS selection using Zymoprep Yeast Plasmid Miniprep II (Zymo Research Cat. No. D2004).

The resulting DNA was used as template in a 20 μL PCR1 reaction with *RAD54*-specific primers using Q5 High-Fidelity DNA Polymerase (NEB, Cat. No. M0491) to amplify a portion of *RAD54* with universal 5′ adaptor overhangs. Product from PCR1 was used directly as 1:20 template in PCR2 reaction with indexed p5/p7 primers. Thermocycler conditions were as follows for PCR1: 98°C for 30 seconds, 98˚C for 10 seconds, 60˚C for 15 seconds, 72˚C for 15 seconds (steps 2-4 repeated 15 times), 72˚C for

two minutes. For PCR2, the annealing temperature was 65˚C and steps 2-4 were repeated 10 times. Barcoded PCR2 reactions were pooled, resolved by 1% agarose gel electrophoresis, and DNA was isolated by Gel Extraction Kit (Qiagen). Paired-end sequencing was performed using an Element AVITI sequencer. Resulting reads were demultiplexed with Bases2Fastq and processed to remove all reads lacking 20-bp sequence upstream and downstream a 9-bp window of interest. This window was extracted, counted, and translated using custom python code. A fold-enrichment score was calculated by comparing the normalized frequency of each amino acid sequence before and after MMS selection. Data was plotted using GraphPad Prism.

Genetic analysis of Rad54 site-directed mutants

Site-directed mutants of pRS415-*RAD54* were made by assembling 300-bp fragments of *RAD54* (IDT eBlocks) with a PCR amplicon consisting of the remainder of pRS415-*RAD54* (oligos MTP364/365). pRS415-*RAD54* and pRS415-*rad54* with site-directed mutations were transformed into strain yECG46 as described above. After a 72-hour incubation at 30˚C, single colonies were restreaked onto SD –Leu and grown for an additional 72 hours at 30˚C. Cultures inoculated from single colonies were grown overnight in liquid SD –Leu, harvested and resuspended in water to a calculated OD_{600} of 10. Cells were 10-fold serially diluted and 4 μL spotted onto SD –Leu supplemented with MMS as indicated in the figures.

Genetic analysis of Hed1 point mutants

HED1 was cloned into the plasmid pYes2 (2μ origin, *URA3* marker, *GAL1* promoter) by generating two fragments by PCR amplification, one from *HED1 S. cerevisiae* W303 genomic DNA with oligos MTP280/MTP281 and another from purified pYes2 with oligos MTP278/MTP279, followed by assembly as above. Site-directed mutants were made by assembling 300-bp fragments of *HED1* (IDT eBlocks) with a PCR amplicon consisting of the remainder of pYes2-*HED1* (oligos MTP366/367).

pYes2-*HED1* and pYes2-*hed1* with site-directed mutations were transformed into strain yECG05 as described above. After a 72-hour incubation at 30˚C, single colonies were restreaked onto SD –Ura and grown for an additional 72 hours at 30˚C. Cultures inoculated from single colonies were grown overnight in liquid SD –Ura and diluted in water to a calculated OD_{600} of 2. Cells were 4-fold serially diluted and 4 μL spotted onto SD –Ura containing either 2% glucose or 2% galactose (Sigma-Aldrich, Cat. No. 91249) supplemented with MMS as indicated in the figures.

Genetic assays with Rad51 PAP mutants

Wild type *RAD51*, along with an additional 500 base pairs upstream and downstream of the gene, was amplified and cloned into the yeast integrative vector pRS406 utilizing PCR-based methods (primers listed in Dataset 1). Point mutations were introduced into the *RAD51* gene via site directed mutagenesis and confirmed with long-read sequencing. The vector constructs (including wildtype, mutants, and empty pRS406) were linearized with NcoI-HF restriction enzyme at the *URA3* locus and transformed into yECG48. Integration at the *URA3* locus was confirmed by PCR. Successfully transformed yeast strains were then subjected to sporulation, and the tetrads were dissected. Haploid yeast strains containing a *RAD51* gene at the *URA3* locus, a deletion of *rad51* at the endogenous locus, and of the *MAT*α mating phenotype were identified and isolated. These single allele *RAD51* haploids were then used for the spot assays. The spot assays were

performed by incubating the strains overnight at 30˚C in liquid YPD media and diluting them to OD_{600} of 1.0 the following day. After additional 10X serial dilutions, the cultures were spotted (4 μL) on freshly poured YPD containing the indicated concentrations of methyl methanesulfonate (MMS; Sigma-Aldrich, Cat. No. 129925) with minimal light exposure. These plates were incubated at 30°C for 2-3 days post spotting and then imaged.

D-loop assays

D-loop formation experiments were performed in HR buffer (30 mM Tris–OAc [pH 7.5], 50 mM KCl, 20 mM MgOAc, 1 mM DTT, 0.2 mg/ml BSA) using an Atto-647N-labeled tailed DNA duplex consisting of a 21 nt overhang that was homologous for a region on the pUC19 plasmid, and a 56 bp dsDNA region that was not homologous to the plasmid as previously described (4)(Dataset 1). Rad51 (WT or triple mutant) (300 nM) was incubated with the tailed duplex DNA (10 nM) in HR buffer supplemented with 10 mM ATP at 30˚C for 15 min. The resulting Rad51-DNA complexes were added to an equal volume of HR buffer containing pUC19 plasmid (9 nM), Rad54 (90 nM), RPA (750 nM) and incubated at 30˚C for 5 min. Reactions were quenched with an equal volume of Stop Buffer (25 mM EDTA, 1% SDS, and 20% glycerol) and then deproteinized by incubation with proteinase K (1 unit) at 37˚C for 20 min. The resulting reaction products were resolved on a 0.9% agarose gel in 1× TAE buffer and detected using a GE Healthcare Life Sciences Typhoon FLA 9500 biomolecular imaging system.

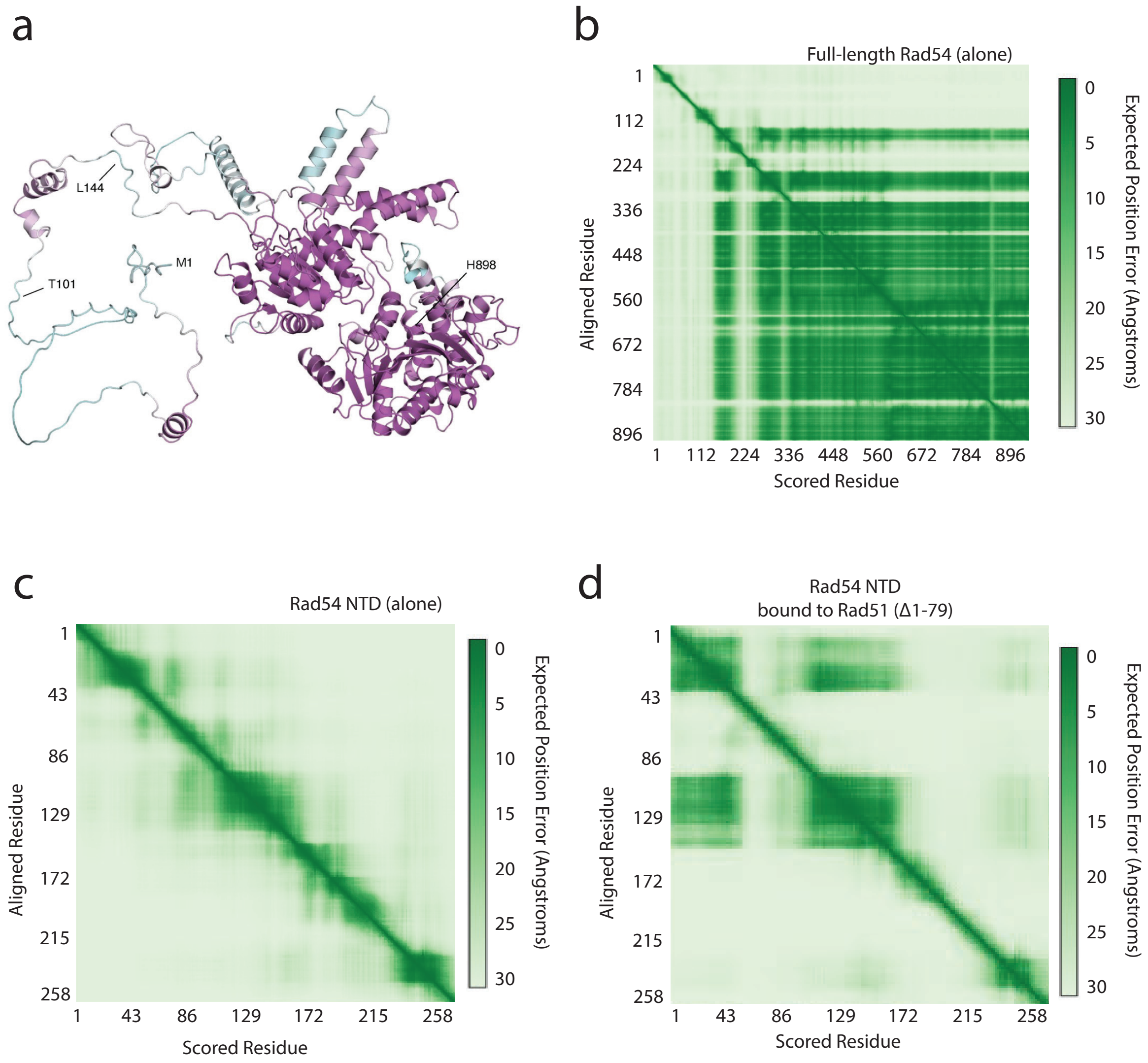

Figure S1. Predicted structure of *S. cerevisiae* Rad54. (A) AlphaFold3 predicted structure of full-length *S. cerevisiae* Rad54 alone. **(B)** Predicted aligned error plot for full-length *S. cerevisiae* Rad54 alone. **(C)** Predicted aligned error plot for *S. cerevisiae* Rad54-NTD alone**. (D)** Predicted aligned error plot for *S. cerevisiae* Rad54-NTD bound to the Rad51 dimer. Only the Rad54-NTD region is shown for the comparison to Panel C.

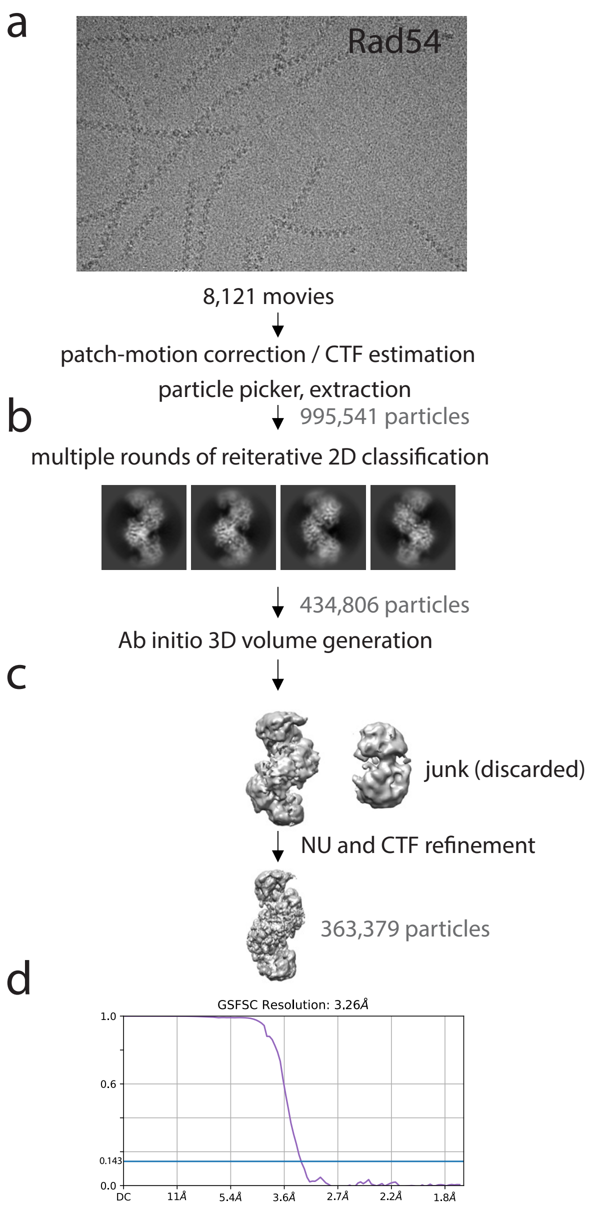

Figure S2. Cryo-EM data processing pipeline for Rad54-Rad51 nucleoprotein filament. **(A)** Representative micrograph used for the Rad51-Rad54 nucleoprotein filament data processing. **(B)** Representative 2D classes selected for 3D classification. **(C)** 3D map generation and refinements. **(D)** Fourier shell correlation curve of the final electron density map.

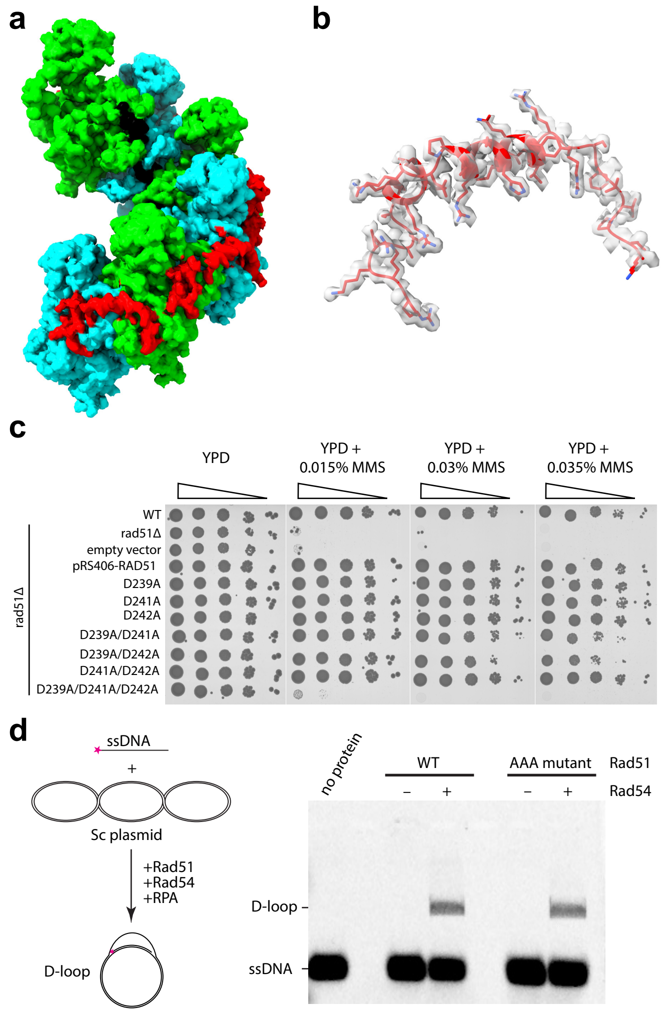

Figure S3. Analysis of the Rad51 interaction domain from Rad54. (A) CryoEM density map of the Rad51-Rad54 nucleoprotein filament. Rad51 is shown in alternative cyan and light green, the Rad54 peptide is red, and the ssDNA is shown in black. **(B)** Fitting of the Rad54 peptide into the CryoEM density map. The CryoEM density map of the Rad54 peptide is shown with transparent surface (gray) and the atomic model of the Rad54 peptide is shown as a cartoon and stick model in red. **(C)** Spot assays for single, double, and triple mutants within the Rad51 protruding acidic patch (PAP) motif on media containing the indicated concentrations of MMS. Note, all spot assays were repeated in triplicate. **(D)** Schematic of the *in vitro* D-loop assay using a fluorescently tagged ssDNA substrate and a supercoiled plasmid (left panel) and a D-loop assay showing that the Rad51 PAP triple mutant (D239A, D241A, D242A; denoted as AAA in the figure) retains Rad54-dependent D-loop activity.

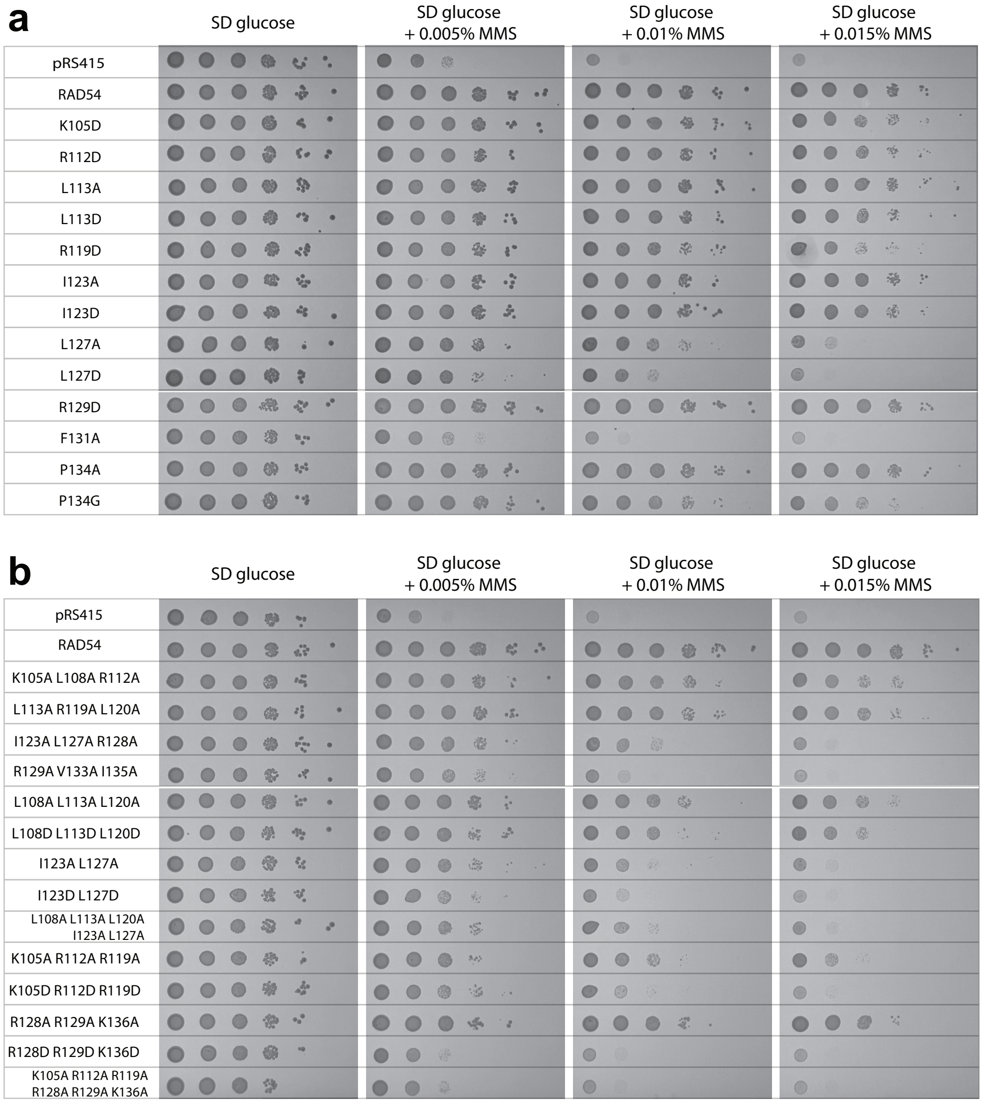

Figure S4. Genetic assays with *rad54* point mutants. (A) Spot assays for selected *rad54* single point mutants on media containing the indicated concentrations of MMS. **(B)** Spot assays for *rad54* harboring multiple mutations on media containing the indicated concentrations of MMS. Spot assays are representative of n=3 experiments.

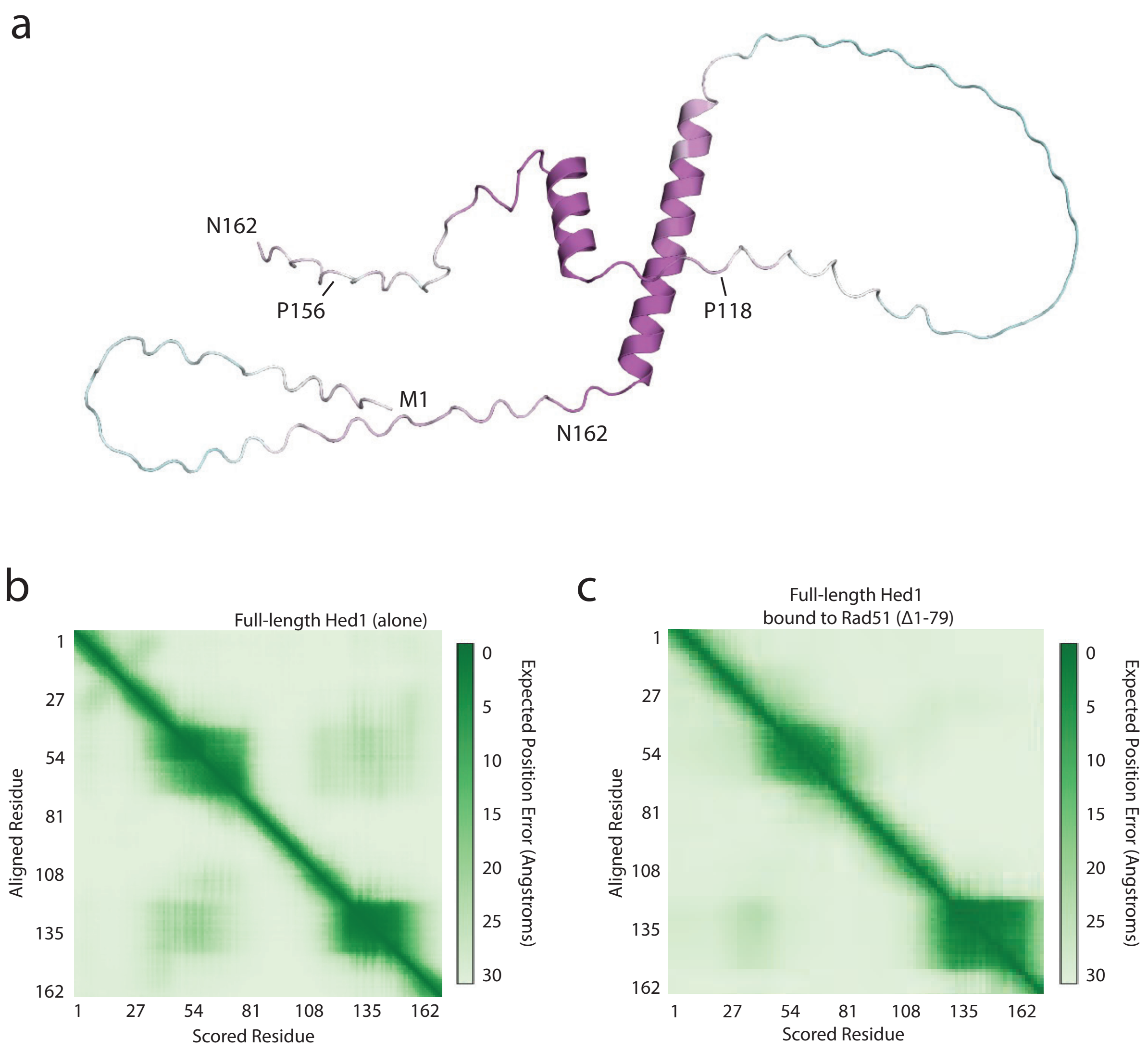

Figure S5. Predicted structure of *S. cerevisiae* Hed1. (A) AlphaFold3 predicted structure of full-length *S. cerevisiae* Hed1 alone. **(B)** Predicted aligned error plot for full-length *S. cerevisiae* Hed1 alone. **(C)** Predicted aligned error plot for full-length Hed1 bound to the Rad51 dimer. Only the Hed1 region is shown for the comparison to Panel B.

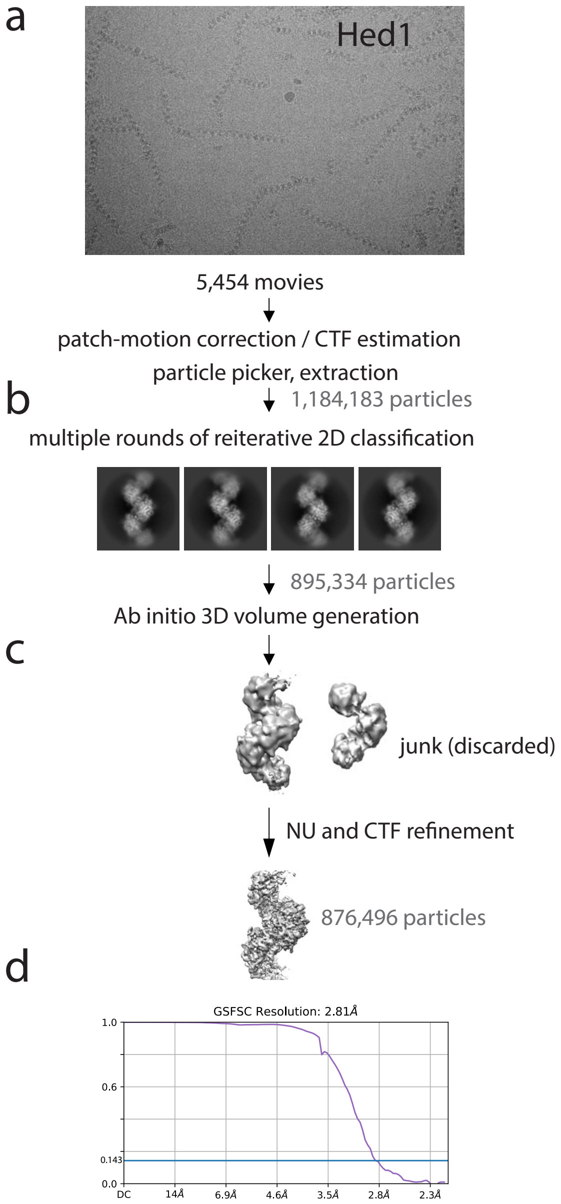

Figure S6. Cryo-EM data processing pipeline for Hed1-Rad51 nucleoprotein filament. **(A)** Representative micrograph used for the Rad51-Hed1 nucleoprotein filament data processing. **(B)** Representative 2D classes selected for 3D classification. **(C)** 3D map generation and refinements. **(D)** Fourier shell correlation curve of the final electron density map.

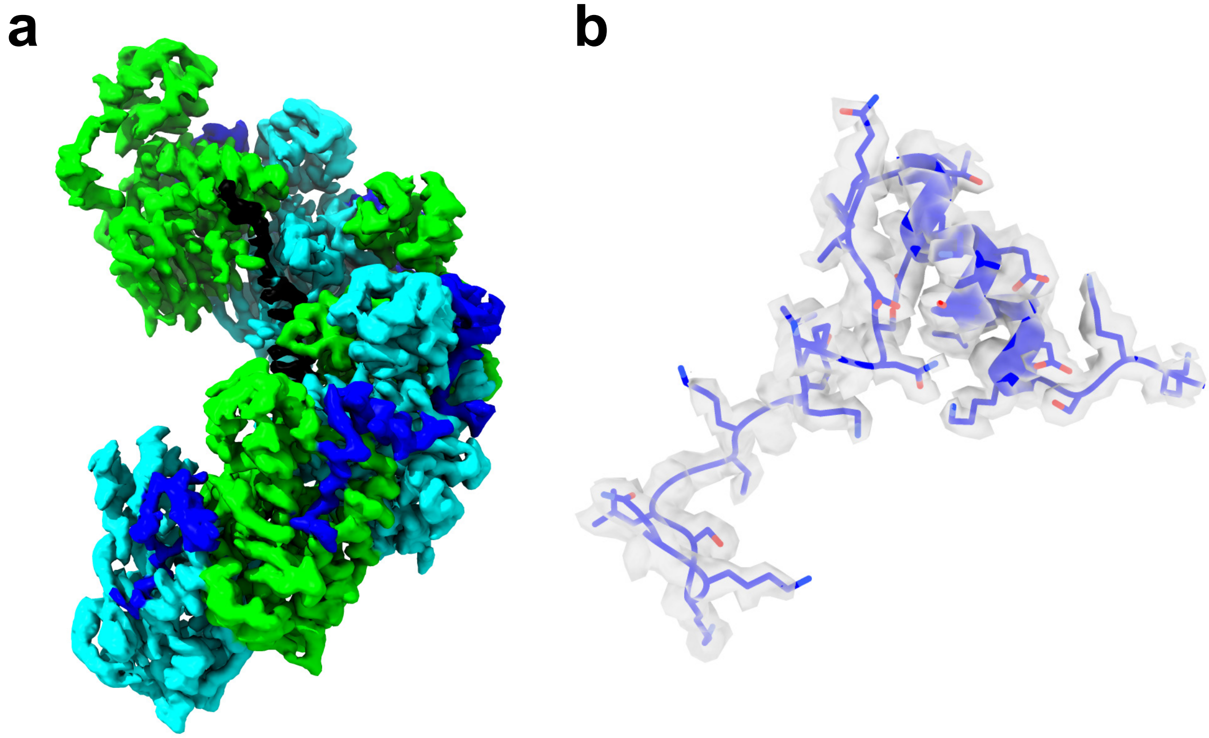

Figure S7. Electron density for the Rad51 interaction domain from Hed1. (A) CryoEM density map of the Rad51-Hed1 nucleoprotein filament. Rad51 is shown in alternating cyan and light green, the Hed1 peptide is dark blue, and the ssDNA is shown in black. **(B)** Fitting of the Hed1 peptide into the CryoEM density map. The CryoEM density map of the Rad54 peptide is shown with transparent surface (gray) and the atomic model of the Rad54 peptide is shown as a cartoon and stick model in blue.

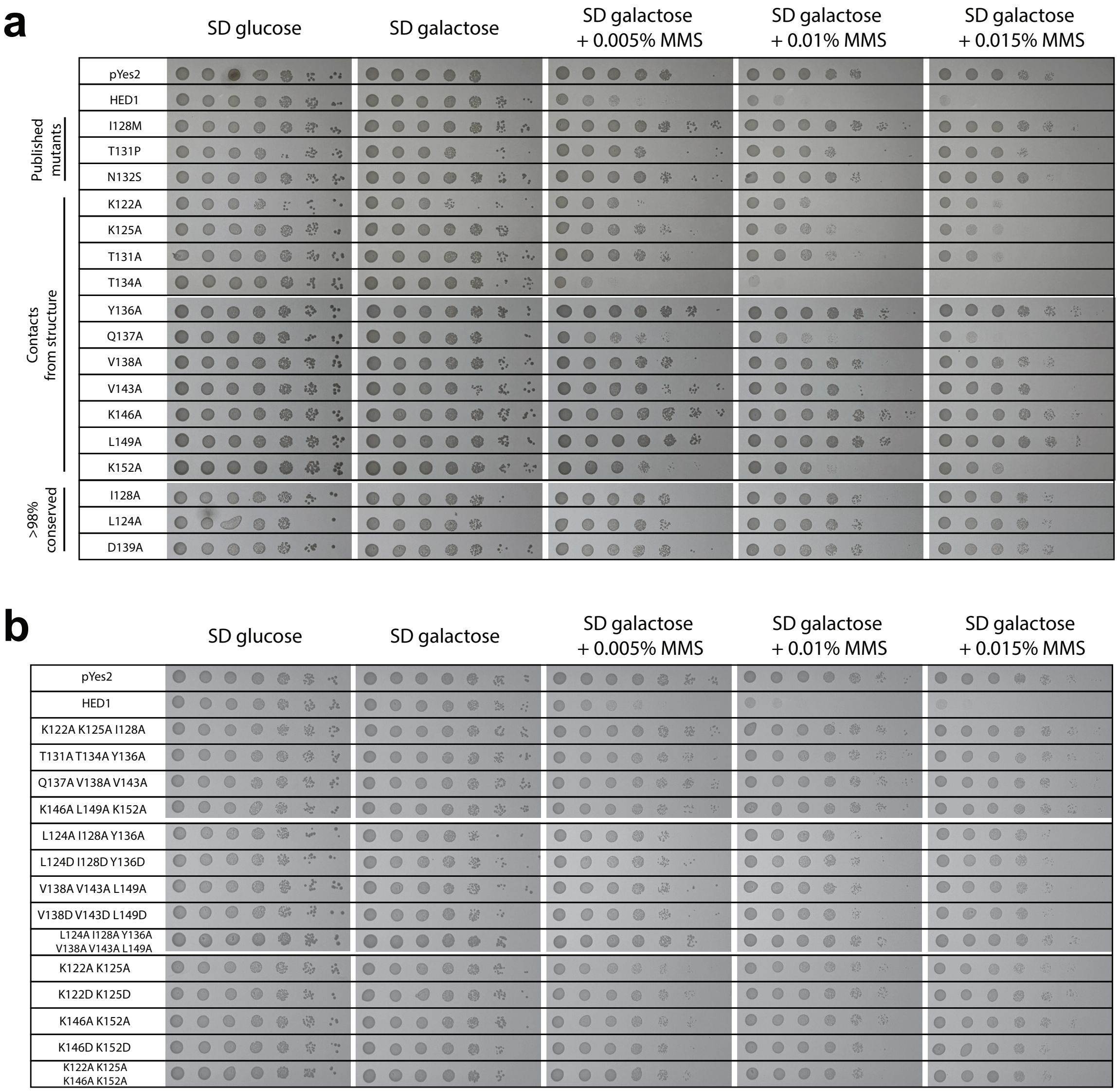

Figure S8. Genetic assays with *hed1* point mutants. (A) Spot assays for selected *hed1* single point mutants on media containing the indicated concentrations of MMS. **(B)** Spot assays for *hed1* harboring multiple mutations on media containing the indicated concentrations of MMS. In these assays, Hed1 expression was suppressed in the presence of glucose, whereas Hed1 expression was induced by the inclusion of galactose in the growth media. Spot assays are representative of n=3 experiments.

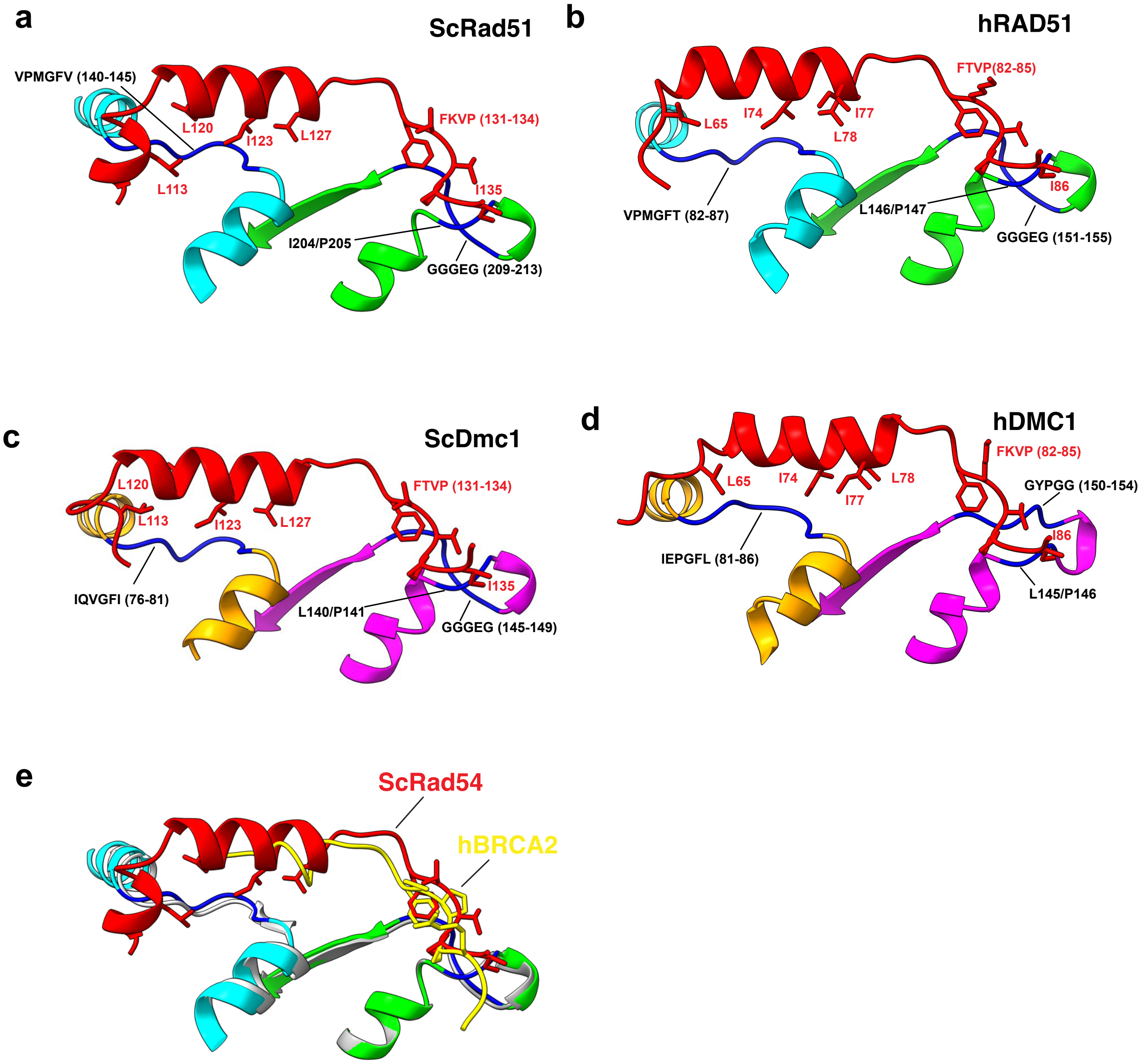

Figure S9. Conservation of the Rad54 interaction with different eukaryotic recombinases. (A) CryoEM structure of the *S. cerevisiae* Rad51 interaction motif from Rad54 (in red) bound to two adjacent Rad51 monomers (cyan and green). **(B)** AlphaFold3 prediction of the Rad51 interaction motif from human RAD54 bound to two adjacent human RAD51 monomers (cyan and green). **(C)** AlphaFold3 prediction of the Rad51 interaction motif from *S. cerevisiae* Rad54 bound to two adjacent *S. cerevisiae* Dmc1 monomers (orange and magenta). **(D)** AlphaFold3 prediction of the Rad51 interaction motif from human RAD54 bound to two adjacent human DMC1 monomers (orange and magenta). Conserved regions of contact are indicated in all panels. **(E)** Overlay of the CryoEM structure for *S. cerevisiae* Rad51 interaction motif from Rad54 (in red) bound to two adjacent Rad51 monomers (cyan and green) with human RAD51 (in gray) bound to the TR2 motif from BRCA2 (in yellow; PDB accession codes 8PBC)(6).

Table S1. CryoEM parameters for the Rad51-Rad54 and Rad51-Hed1 nucleoprotein filaments.

	Rad51_Rad54<br>PDB: 9E6L<br>(EMD-47572)	Rad51_Hed1<br>PDB: 9E6N<br>(EMD-47573)
**Data collection and processing**		
Microscope	Titan Krios	Titan Krios
Voltage (keV)	300	300
Detector	K3	K3
Magnification	105,000	85,000
Voltage (kV)	300	300
Electron exposure ($e^-/Å^2$)	59.51	51.21
Defocus range (μm)	-0.8 to -2.5	-0.8 to -2.5
Pixel size (Å)	0.844	1.083
Initial particles picked	995,541	1,184,183
Final particles used	363,379	876,496
Map resolution (Å)<br>FSC threshold	3.3<br>0.143	2.8<br>0.143
Map resolution range (Å)	3.3-3.7	2.8-3.3
**Refinement**		
Model resolution (Å)<br>FSC threshold	3.3<br>0.143	2.8<br>0.143
Model composition<br>Non-hydrogen atoms<br>Protein residues<br>Ligands	 16,562<br>2,092<br>ATP, MG	 16,759<br>2,115<br>ATP, MG
R.m.s. deviations<br>Bond lengths (Å)<br>Bond angles (°)	 0.002<br>0.453	 0.003<br>0.467
Validation<br>MolProbity score<br>Clash score<br>Rotamer outliers (%)	 1.27<br>5.14<br>0	 1.25<br>4.81<br>0
Ramachandran plot<br>Favored (%)<br>Allowed (%)<br>Outliers (%)	 98.69<br>1.26<br>0	 98.42<br>1.58<br>0

Table S2. Accession codes. Included the Protein Data Bank (PDB) and Electron Microscopy Data Bank (EMDB) accession codes for the Rad54-Rad51-ssDNA and Hed1-Rad51-ssDNA structures, the URL for accessing original code on Github, and the NCBI Sequence Read Archive (SRA) BioProject ID for accessing the deep sequencing data.

Data	Accession information	
Rad51-Rad54 complex	PDB: 9E6L	EMD-47572
Rad51-Hed1 complex	PDB: 9E6N	EMD-47573
Github code	https://github.com/michaeltpetassi/Rad54Hed1_2025	
NCBI Sequence Read Archive	BioProject ID : PRJNA1235687	

SUPPORTING REFERENCES

1. E. W. Sayers *et al.*, Database resources of the national center for biotechnology information. *Nucleic Acids Res* **50**, D20-d26 (2022).

2. L. Fu, B. Niu, Z. Zhu, S. Wu, W. Li, CD-HIT: accelerated for clustering the next-generation sequencing data. *Bioinformatics* **28**, 3150-3152 (2012).

3. R. C. Edgar, MUSCLE: multiple sequence alignment with high accuracy and high throughput. *Nucleic Acids Res* **32**, 1792-1797 (2004).

4. J. B. Crickard, C. J. Moevus, Y. Kwon, P. Sung, E. C. Greene, Rad54 Drives ATP Hydrolysis-Dependent DNA Sequence Alignment during Homologous Recombination. *Cell* **181**, 1380-1394.e1318 (2020).

5. J. Abramson *et al.*, Accurate structure prediction of biomolecular interactions with AlphaFold 3. *Nature* **630**, 493-500 (2024).

6. R. Appleby, L. Joudeh, K. Cobbett, L. Pellegrini, Structural basis for stabilisation of the RAD51 nucleoprotein filament by BRCA2. *Nat Commun* **14**, 7003 (2023).
